# Molecular Diversity of Glutamatergic and GABAergic Synapses from Multiplexed Fluorescence Imaging

**DOI:** 10.1523/ENEURO.0286-20.2020

**Published:** 2021-01-15

**Authors:** Eric Danielson, Karen Perez de Arce, Beth Cimini, Eike-Christian Wamhoff, Shantanu Singh, Jeffrey R. Cottrell, Anne E. Carpenter, Mark Bathe

**Affiliations:** 1 Department of Biological Engineering, MIT, Cambridge 02142, MA; 2 Stanley Center for Psychiatric Research, Broad Institute of MIT and Harvard, Cambridge 02142, MA; 3 Imaging Platform, Broad Institute of MIT and Harvard, Cambridge 02142, MA

**Keywords:** machine learning, multiplexed imaging, synaptic plasticity, synaptic scaling

## Abstract

Neuronal synapses contain hundreds of different protein species important for regulating signal transmission. Characterizing differential expression profiles of proteins within synapses in distinct regions of the brain has revealed a high degree of synaptic diversity defined by unique molecular organization. Multiplexed imaging of *in vitro* rat primary hippocampal culture models at single synapse resolution offers new opportunities for exploring synaptic reorganization in response to chemical and genetic perturbations. Here, we combine 12-color multiplexed fluorescence imaging with quantitative image analysis and machine learning to identify novel synaptic subtypes within excitatory and inhibitory synapses based on the expression profiles of major synaptic components. We characterize differences in the correlated expression of proteins within these subtypes and we examine how the distribution of these synapses is modified following induction of synaptic plasticity. Under chronic suppression of neuronal activity, phenotypic characterization revealed coordinated increases in both excitatory and inhibitory protein levels without changes in the distribution of synaptic subtypes, suggesting concerted events targeting glutamatergic and GABAergic synapses. Our results offer molecular insight into the mechanisms of synaptic plasticity.

## Significance Statement

An immense number of proteins are present at synapses to regulate synaptic function. Recent efforts characterizing synaptic protein expression patterns suggest their differential expression gives rise to diverse synapse subpopulations. In this work, we use multiplexed fluorescence imaging with advanced image analysis to detect and quantify protein levels using CellProfiler. We apply our technique to develop a robust approach for unbiased synaptic subtype identification based on protein expression profiles using uniform manifold approximation and projection (UMAP) dimensional reduction and hierarchical density-based spatial clustering of applications with noise (HDBSCAN) clustering. Finally, we apply this approach to examine synaptic diversity in cultured hippocampal neurons and examine the molecular events of 11 proteins at excitatory and inhibitory synapses following synaptic scaling.

## Introduction

Synapses contain complex proteomes that organize into multiprotein signaling complexes (Husi et al., 2000; Collins et al., 2006). There appears to be a high degree of diversity in the expression, stoichiometry, and organization of these proteins across scales from individual synapses to the entire brain (Emes and Grant, 2012; Roy et al., 2018; Zhu et al., 2018). While it is unknown how many classes of synapses exist, there are over 1000 genes that encode synaptic proteins (Husi et al., 2000; Peng et al., 2004; Collins et al., 2005, 2006; Emes et al., 2008; Bayés et al., 2011, 2012, 2017; Emes and Grant, 2011; Distler et al., 2014). The differential expression of these proteins across distinct brain regions, as well as differential spatial-temporal expression during development, suggest significant synaptic diversity. Immunofluorescence labeling of two differentially expressed excitatory scaffolding proteins was used to examine synaptic diversity throughout the mouse brain (Zhu et al., 2018) however, to date how synaptic diversity is affected by synaptic scaling has not been investigated. Synaptic scaling maintains neuronal homeostasis via global changes in expression of multiple synaptic proteins targeting both excitatory and inhibitory synapses to prevent runaway neuronal excitability (Turrigiano et al., 1998; Turrigiano and Nelson, 2000, 2004; Turrigiano, 2008, 2012). Examination of the entire molecular composition of individual synapses using techniques that can survey the entire proteome, such as mass spectrometry, would be ideal for characterizing synapses. However, synapses are ∼2 μm in size; and while advances in mass spectrometry-based imaging have achieved 1-μm resolution (Zavalin et al., 2015), the majority of commercial matrix**-**assisted laser desorption/ionization (MALDI) mass spectrometers are not sufficiently accurate for examination of individual synapses. Hence, microscopy techniques, such as immunofluorescence, remains the optimal technique for the examination of individual synapses. However, conventional fluorescence microscopy is generally limited to four channels as a result of the maximal spectral resolution of organic and biomolecular fluorophores. This limitation presents a challenge to comprehensive analysis of synaptic architecture because of the large number of different protein species within each synapse.

Multiplexed imaging techniques are particularly useful for studying neuronal synapses, and investigating the coordinated assembly of dozens to hundreds of distinct proteins involved in synaptic development, function, and plasticity. Probe-based imaging for sequential multiplexing (PRISM) is a recently introduced multiplexed imaging technique that uses single-stranded DNA (ssDNA)-conjugated antibodies or peptides with complementary fluorescently labeled single-stranded locked nucleic acid (ssLNA) imaging probes to sequentially visualize multiple synaptic targets *in situ* (Guo et al., 2019). With this approach, the affinity of the ssLNA imaging probes is salt dependent, allowing multiple synaptic targets to be imaged within the same sample through sequential rounds of imaging first in physiological salt buffer, followed by rapid imaging-strand removal in low-salt buffer. This method of imaging prevents the neuronal sample disruption that occurs with alternative multiplex imaging techniques (Gerdes et al., 2013; Lin et al., 2015). PRISM has been used to quantify changes in synaptic protein levels, co-expression profiles, and synapse-subtype compositions following blockade of action potentials with tetrodotoxin (TTX) treatment (Guo et al., 2019). This analysis revealed that TTX induces a coordinated reorganization of excitatory presynaptic and postsynaptic proteins. However, structural plasticity in response to chronic activity changes occurs in both excitatory and inhibitory synapses which was not addressed in that study.

Here, we used PRISM to measure homeostatic structural changes of subcellular compartments of rat hippocampal neurons, simultaneously discriminating between excitatory and inhibitory terminals. In addition, we created an automated imaging and analysis pipeline in the open-source bioimage analysis software CellProfiler that identifies and characterizes synaptic puncta from multiplexed images to compare changes in protein levels that occur in excitatory and inhibitory synapses. Using this computational framework, we identified six distinct classes of synapses and found differential regulation of synapsin1 at both excitatory and inhibitory synapses in response to TTX treatment. However, while we found increased inhibitory presynaptic protein levels following activity suppression, TTX treatment did not affect synaptic diversity in 21 d *in vitro* (DIV21) neurons. These results broaden our knowledge of the molecular events that occur during synaptic plasticity.

## Materials and Methods

### Primary rat neuronal cultures

Procedures for rat neuronal culture were reviewed and approved for use by the Broad Institutional Animal Care and Use Committee. For hippocampal neuronal cultures, 8–12 Embryonic Day 18 embryos were collected from each pregnant Sprague Dawley rats killed by CO_2_ (Taconic) processed separately. Embryo hippocampi were dissected in 4°C Hibernate E supplemented with 2% B27 supplements and 100 U/ml penicillin/strep (Thermo Fisher Scientific). Hippocampal tissues were digested in Hibernate E containing 20 U/ml papain, 1 mm L-cysteine, 0.5 mm EDTA (Worthington Biochem), and 0.01% DNase (Sigma-Aldrich) for 8 min and stopped with 0.5% ovomucoid trypsin inhibitor (Worthington Biochem) and 0.5% bovine serum albumin (BSA; Sigma-Aldrich). Neurons were plated at a density of 15,000 cells/well onto poly-d-lysine-coated, black-walled, thin-bottomed 96-well plates (Greiner Bio-One). Neurons were seeded and maintained in NbActiv1 (BrainBits) at 37°C in 95% air with a 5% CO_2_ humidified incubator for 19 d before use. TTX-treated neurons were treated on day 19 with 1 μm TTX (Tocris Bioscience) for 48 h then harvested for immunostaining. All procedures involving animals were in accordance with the *National Institutes of Health Guide for the Care and Use of Laboratory Animals*.

### Immunostaining for LNA-PRISM

Following TTX-treatment cells were fixed in 4% paraformaldehyde in and 4% wt/vol sucrose in PBS for 15 min at room temperature and then permeabilized with 0.25% Triton X-100 in PBS. Permeabilized cells were incubated in 50 μg/ml RNase A (Thermo Fisher Scientific) and 230 U/ml RNase T1 (Thermo Fisher Scientific) in PBS at 37°C for 1 h to reduce the fluorescent background caused by ssLNA-RNA binding and subsequently washed three times with PBS. Cells were then blocked for 1 h at room temperature with 5% BSA in PBS. The first round of primary staining was performed using unconjugated primary antibodies diluted in the regular blocking buffer: MAP2, VGLUT1, postsynaptic density-95 (PSD-95), NR2B, and gephyrin. Cells were incubated in primary antibodies overnight at 4°C, washed three times with PBS, and then incubated in the nuclear blocking buffer [5% BSA and 1 mg/ml salmon sperm DNA (Sigma-Aldrich) in PBS] for 1 h at room temperature. The first round of secondary staining antibodies was diluted in the nuclear blocking buffer and incubated for 1 h at room temperature. Antibodies used in the first round of secondary staining was: goat-anti-chicken-Alexa Fluor 488, goat-anti-guinea pig-Alexa Fluor 555 and goat-anti-rabbit-p1, goat-anti-mouse-p12, and goat-anti-rat-p7. Cells were washed three times with PBS, postfixed for 15 min with 4% paraformaldehyde and 4% wt/vol sucrose in PBS. Cells were washed three times with PBS and incubated again in the nuclear blocking buffer for 30 min at room temperature. The second round of primary antibodies were incubated overnight at 4°C in the following primary antibody/peptide solution diluted in the nuclear blocking buffer using: phalloidin-p2, vGAT-p3, cortactin-p4, SHANK3-p6, bassoon-p8, synapsin1-p9, and homer-1b/c-p10. Cells were then washed three times with PBS then DAPI stained for 15 min. See Table 1 for detailed antibody information and antibody conjugation method.

**Table 1:** Antibody information

Antibody target	Vendor	Catalog number	Species and clonality	Conjugation strategy	Docking strand sequence	Working concentration (μg/ml)
PSD-95	Cell Signaling Technology	3450	Rabbit monoclonal	−	−	0.1
Bassoon	Enzo Life Sciences	ADI-VAM-PS003	Mouse monoclonal	SMCC	p8	4
vGAT	Synaptic Systems	131011	Mouse monoclonal	SMCC	p3	10
Gephyrin	Synaptic Systems	147208	Rat chimeric	−	−	3
MAP2	Novus Biologicals	NB300-213	Chicken polyclonal	−	−	
Phalloidin	Bachem	H-7634	−	SMCC	p2	50
Cortactin	Millipore	05–180	Mouse monoclonal	SMCC	p4	20
Synapsin1	Santa Cruz	sc-7379	Goat polyclonal	SMCC	p9	3
SHANK3	Santa Cruz	sc-30193	Rabbit polyclonal	SiteClick	p6	7.4
Homer-1b/c	Santa Cruz	sc-20807	Rabbit polyclonal	SMCC	p10	4
NR2B	NeuroMab	75–097	Mouse monoclonal	−	−	10
Anti-rabbit secondary	Life Technologies	A16126	Goat polyclonal	SMCC	p1	3
Anti-mouse secondary	Life Technologies	A16068	Goat polyclonal	SMCC	p12	3
Anti-rat secondary	Invitrogen	A18873	Goat polyclonal	SMCC	p7	8
MAP2	Abcam	Ab5392	Chicken polyclonal	−	−	9.5
vGlut1	Synaptic Systems	135304	Guinea pig polyclonal	−	−	1:400 (dilution)
Alexa Fluor 488 anti- chicken secondary	Thermo Fisher	A11039	Goat polyclonal	−	−	4
Alexa Fluor 555 anti- guinea pig secondary	Thermo Fisher	A21435	Goat polyclonal	−	−	4

All antibodies and the working concentration used in this study are listed with the company name catalog and conjugation style.

### Multiplexed confocal imaging of neurons using LNA-PRISM

LNA-PRISM imaging was performed using the Opera Phenix High-Content Screening System (PerkinElmer) equipped with a fast laser-based autofocus system, high NA water immersion objective (63×, numerical aperture = 1.15), two large format scientific complementary metal-oxide semiconductor (sCMOS) cameras and spinning disk optics; 405-, 488-, and 561-nm wavelength laser lines were used as excitation for DAPI, MAP2, and vGlut1 channels, respectively. PRISM images were acquired using a 640-nm laser (40 mW), sCMOS camera with 1- to 2-s exposure time, and effective pixel size of 187 nm. Imaging probe was freshly diluted to 10 nm in imaging buffer (500 mm NaCl in PBS, pH 8) immediately before imaging. Neurons were incubated with imaging probes for 5 min and then washed twice with imaging buffer to remove unbound probe. See Table 2 for LNA imager probe sequences. For each field of view, a stack of three images was acquired with an axial step-size of 0.5 μm. At least five lateral fields of view were imaged per replicate of the six replicates per condition, un-treated and TTX treated. Following each round of imaging, cells were washed two times with wash buffer (0.01 × PBS) for 3 min per round.

**Table 2:** Docking strand and imaging probe sequences used in PRISM

Sequence name	Docking strand sequence (5′ to 3′)	ssLNA imaging probe sequence (5′ to 3′)
p1	TTATACATCTA	T*AGAT*G*TATAA
p2	TTATCTACATA	TATGT*A*G*ATAA
p3	TTTCTTCATTA	TAAT*G*A*AGAAA
p4	TTATGAATCTA	TA*GAT*T*CATAA
p5	Not used in this study	Not used in this study
p6	TTAATTGAGTA	T*A*CTCAATTAA
p7	TTAATTAGGAT	A*T*CCT*AATTAA
p8	TTATAATGGAT	A*T*CC*ATTATAA
p9	TTTAATAAGGT	A*CC*T*TATTAAA
p10	TTATAGAGAAG	C*T*TC*TCTATAA
p11	Not used in this study	Not used in this study
p12	TTATAGTGATT	A*ATC*A*CTATAA

LNA substitutions are indicated with * following the LNA nucleotide.

### Image analysis

CellProfiler (McQuin et al., 2018) is a popular image analysis tool containing numerous image segmentation methods and analysis tools. The pipeline can be divided into three main stages. In the first stage the images are imported, aligned and uneven illumination is corrected. The output from this first stage of the pipeline is the aligned tiff images with the corrected background. These images serve as a quality control checkpoint to ensure correct image alignment and illumination correction. The second stage of the pipeline performs image segmentation on the images to define and locate the nuclei, dendrites and puncta from each round of imaging. The pipeline offers users the ability to easily customize key aspects of the analysis methodology such as illumination correction and thresholding without any source code modifications. This stage of the pipeline produces binary images of the object segmentation and also the gray-scale images of the puncta channels following application of the white-top hat filters used to enhance puncta segmentation. These images serve as helpful guides to ensure optimal image segmentation. The final stage of the pipeline groups all of the segmented objects into synapses, based on the level of overlap with the synapsin1 objects, nuclear masks and the dendritic masks. For our analysis puncta were considered non-synaptic if the puncta overlapped the nuclei region, were outside of the dendrite object masks or did not overlap with synapsin1. Postsynaptic objects with at least 6.25% overlap with synapsin1 were considered synaptic. Presynaptic objects were considered synaptic if the overlap was at least 50%. The final stage of the pipeline outputs binary images of the synaptic objects and multiple csv files containing the quantification of the synaptic objects (Extended Data Fig. 1-2). For thresholding, the RobustBackground method was used to choose a threshold value for each synaptic target. This method was first applied to all images corresponding to the untreated group. The resulting threshold values were then averaged to produce a single threshold for each target, which was applied to both the untreated and TTX-treated group to ensure that all images were segmented identically. Only puncta with equivalent diameters between 3 and 15 pixels were included in the analysis. The equivalent diameter is the is the diameter of a circle with the same area as the measured object. Synapses were defined as excitatory when only vGlut1 puncta were present and as inhibitory when only vGAT puncta were present. For quality control of clustering, we isolated and plotted synapses from individual replicates separately to ensure identified clusters were not artifacts resulting from differences in staining or image intensity among the image sets (Extended Data Fig. 5-1).

### PRISM antibody validation

Conjugation of antibodies and peptides with ssDNA may alter antibody affinity and specificity. In order to confirm that ssDNA-conjugated antibodies and peptides were unperturbed by conjugation, extensive validation was performed for each synaptic and cytoskeletal target. Specifically, for each target a mixture containing both conjugated and unconjugated antibodies was applied to neuronal samples to verify that staining patterns of the unconjugated and conjugated antibodies were the same.

### Code accessibility

The automated CellProfiler synaptic protein analysis pipeline is available as Extended Data 1.

10.1523/ENEURO.0286-20.2020.ed1 Extended Data 1 CellProfiler multiplexed image analysis pipeline. The included image analysis pipeline automates the detection and quantification of synaptic puncta from multiplexed images. Download Extended Data 1, ZIP file.

### Statistical analysis

All data are presented using their mean ± 95% confidence interval with *N *=* *6 independent neuronal culture replicates for untreated and *N *=* *6 neuronal culture replicates for TTX-treated samples. The mean intensity of each experimental condition was normalized to the mean intensity of the untreated wells within the respective plate. For each individual replicate, five to nine images were collected. To ensure consistent, uniform data collection across each of the 6 imaged replicates corresponding to each treatment condition, 2000 individual synapses were randomly subsampled from each replicate and combined in the final dataset to ensure that each replicate contributed equally to the final results. During imaging, a mechanical failure occurred while imaging one of the TTX-treated replicates so that it was necessary to eliminate this replicate from the final dataset, resulting in *N *=* *6 untreated and *N *=* *5 TTX-treated samples including in the final data and statistical analyses. All statistical analysis was performed using R statistical environment. Two-tailed permutation *t* tests were performed by calculating the mean difference from 5000 reshuffles of the untreated and TTX-treated samples. The *p* values represent the likelihood of observing the reported effect size, if the null hypothesis of zero difference is true. For all data where *t* test was performed effect size was also calculated using effsize R package (Torchiano, 2016). Effect size confidence intervals were calculated using random resampling (*n* = 5000) of mean integrated intensity measurements from each replicate (*n* = 6.5; untreated, TTX) using boot package in R (Davison and Hinkley, 1997). All confidence intervals are bias-corrected and accelerated. Plots created using ggridges package in R (Wilke, 2018). Power analysis performed using pwr.t2n.test in pwr package in R (Champely, 2020). Barplots were created using ggplot2 and ggpubr packages in R (Wickham, 2016; Kassambara, 2019). Uniform manifold approximation and projection (UMAP) was performed using the umap package in R stats (Konopka, 2019). Columns containing missing data (NA) were removed. Feature reduction was performed using findCorrelation in caret package of R stats (Wing et al., 2019). The remaining columns following feature reduction are listed in Table 3. Remaining data were centered using the scale function of R Stats before using the data as input for UMAP. The resulting UMAP object was used to transform all remaining data. Hierarchical density-based spatial clustering of applications with noise (HDBSCAN) was performed on umap output using hdbscan module from python with min_cluster_size = 100 (McInnes et al., 2017). Data considered as noise from HDBSCAN was removed from the plot. Scatterplots were generated using ggplot2 and ggpubr. Heatmaps were created using gplots R package (Warnes et al., 2020). Individual correlation matrices were generated for each biological replicate within each UMAP group and then averaged to produce a final representative correlation matrix for each UMAP group. Correlation values between −0.4 and 0.4 obscured to highlight strong correlations.

**Table 3:** Specific features from CellProfiler used as input for UMAP

Metric	Region of interest source	Target used for measurement
Compactness	Synapsin1	Synapsin1
Eccentricity	Synapsin1	Synapsin1
Euler number	Synapsin1	Synapsin1
Extent	Synapsin1	Synapsin1
Form factor	Synapsin1	Synapsin1
Major axis length	Synapsin1	Synapsin1
Maximum radius	Synapsin1	Synapsin1
Mean radius	Synapsin1	Synapsin1
Minor axis length	Synapsin1	Synapsin1
Orientation	Synapsin1	Synapsin1
Perimeter	Synapsin1	Synapsin1
Solidity	Synapsin1	Synapsin1
Puncta number	Synapsin1	Homer-1b/c
Puncta number	Synapsin1	NR2B
Puncta number	Synapsin1	PSD-95
Puncta number	Synapsin1	SHANK3
Puncta number	Synapsin1	Actin
Puncta number	Synapsin1	Cortactin
Puncta number	Synapsin1	vGlut-1
Integrated intensity edge	Synapsin1	DAPI
Integrated intensity edge	Synapsin1	Gephyrin
Integrated intensity	Synapsin1	NR2B
Integrated intensity	Synapsin1	PSD-95
Integrated intensity	Synapsin1	SHANK3
Integrated intensity	Synapsin1	Actin
Integrated intensity	Synapsin1	Synapsin1
Integrated intensity	Synapsin1	vGlut-1
Lower quartile intensity	Synapsin1	Synapsin1
MAD intensity	Synapsin1	DAPI
MAD intensity	Synapsin1	Gephyrin
MAD intensity	Synapsin1	Homer-1b/c
MAD intensity	Synapsin1	MAP2
MAD intensity	Synapsin1	PSD-95
MAD intensity	Synapsin1	SHANK3
MAD intensity	Synapsin1	Actin
MAD intensity	Synapsin1	Bassoon
MAD intensity	Synapsin1	Cortactin
MAD intensity	Synapsin1	Synapsin1
MAD intensity	Synapsin1	vGAT
Mass displacement	Synapsin1	DAPI
Mass displacement	Synapsin1	Gephyrin
Mass displacement	Synapsin1	Homer-1b/c
Mass displacement	Synapsin1	MAP2
Mass displacement	Synapsin1	NR2B
Mass displacement	Synapsin1	PSD-95
Mass displacement	Synapsin1	SHANK3
Mass displacement	Synapsin1	Actin
Mass displacement	Synapsin1	Bassoon
Mass displacement	Synapsin1	Cortactin
Mass displacement	Synapsin1	Synapsin1
Mass displacement	Synapsin1	vGlut-1
Mass displacement	Synapsin1	vGAT
Min intensity edge	Synapsin1	Homer-1b/c
Min intensity edge	Synapsin1	NR2B
Min intensity edge	Synapsin1	PSD-95
Min intensity edge	Synapsin1	SHANK3
Min intensity edge	Synapsin1	Actin
Min intensity edge	Synapsin1	Bassoon
Min intensity edge	Synapsin1	Cortactin
Min intensity edge	Synapsin1	vGlut-1
Min intensity	Synapsin1	Gephyrin
Min intensity	Synapsin1	MAP2
Min intensity	Synapsin1	Synapsin1
Min intensity	Synapsin1	vGAT
Std intensity	Synapsin1	Gephyrin
Std intensity	Synapsin1	NR2B
Std intensity	Synapsin1	vGlut-1
Upper quartile intensity	Synapsin1	DAPI
Distance (centroid)	Gephyrin	Gephyrin
Integrated intensity	Gephyrin	Gephyrin
Mass displacement	Gephyrin	Gephyrin
Std intensity	Gephyrin	Gephyrin
Distance (centroid)	Homer-1b/c	Homer-1b/c
Integrated intensity	Homer-1b/c	Homer-1b/c
MAD intensity	Homer-1b/c	Homer-1b/c
Mass displacement	Homer-1b/c	Homer-1b/c
Min intensity edge	Homer-1b/c	Homer-1b/c
Distance (minimum)	NR2B	NR2B
Integrated intensity	NR2B	NR2B
MAD intensity	NR2B	NR2B
Mass dissplacement	NR2B	NR2B
Distance (minimum)	PSD-95	PSD-95
Integrated intensity	PSD-95	PSD-95
MAD intensity	PSD-95	PSD-95
Mass displacement	PSD-95	PSD-95
Min intensity	PSD-95	PSD-95
Distance (minimum)	SHANK3	SHANK3
Integrated intensity	SHANK3	SHANK3
MAD intensity	SHANK3	SHANK3
Mass displacement	Actin	Actin
Distance (centroid)	Actin	Actin
Integrated intensity	Actin	Actin
MAD intensity	Actin	Actin
Mass displacement	Actin	Actin
Distance (centroid)	Bassoon	Bassoon
MAD intensity	Bassoon	Bassoon
Mass displacement	Bassoon	Bassoon
Distance (centroid)	Cortactin	Cortactin
Integrated intensity	Cortactin	Cortactin
MAD intensity	Cortactin	Cortactin
Mass displacement	Cortactin	Cortactin
Min intensity	Cortactin	Cortactin
MAD intensity	vGlut-1	vGlut-1
Mass displacement	vGlut-1	vGlut-1
Min intensity edge	vGlut-1	vGlut-1
Mass displacement	vGAT	vGAT
Min intensity	vGAT	vGAT
Std intensity edge	vGAT	vGAT

The region of interest source is the image that was used to create the regions of interest (the puncta objects). The target used for measurement is the image source that the region of interest was used on. For example, synapsin1 puncta (regions of interest) are always created from synapsin1 images, but measurements using those regions can come from any image source, i.e., we can examine what the signal of vGlut-1, homer-1b/c, etc. is within the regions created by the synapsin1 objects.

## Results

### Characterization of synaptic content from multiplexed images

**Figure 1.**
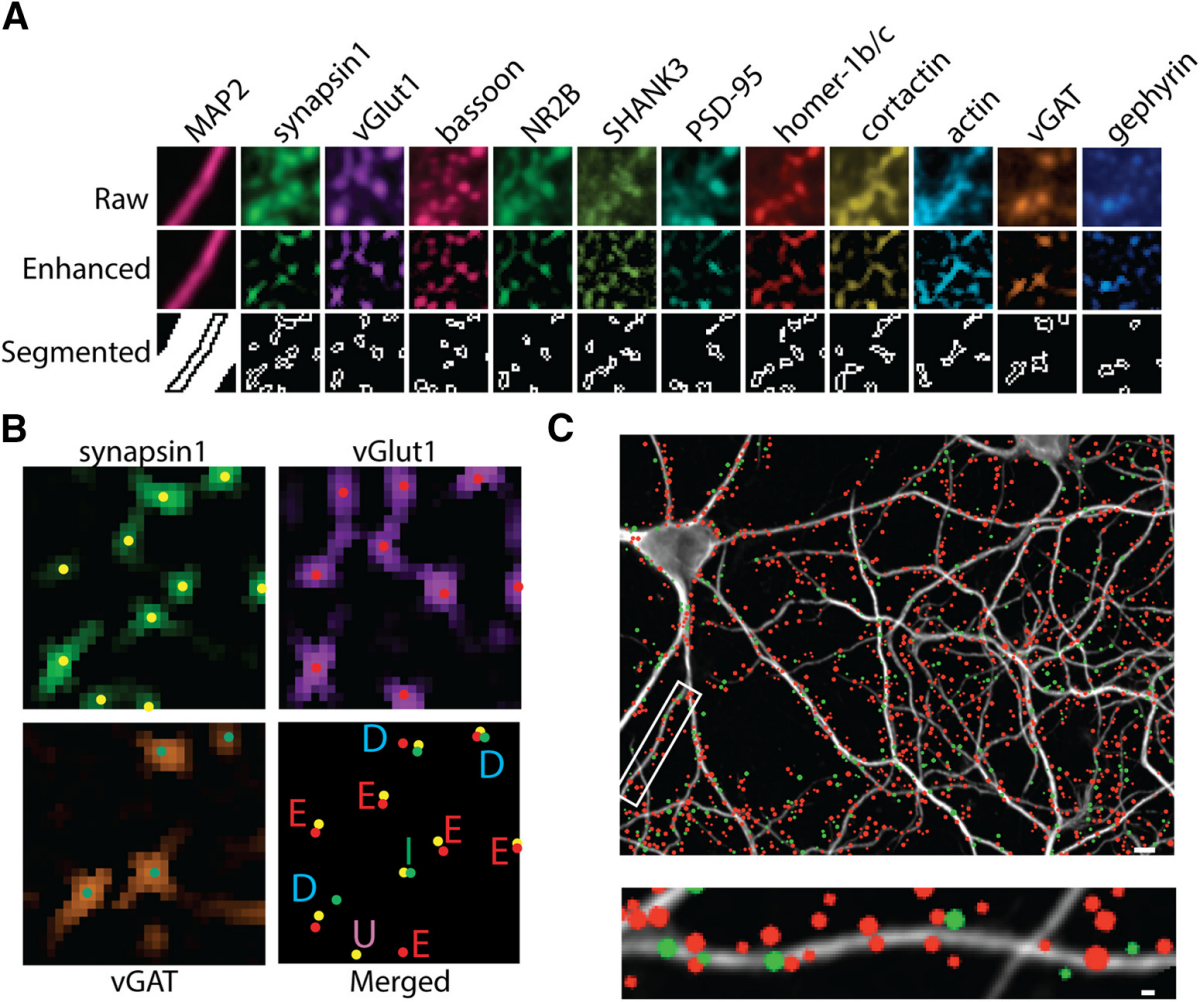
Measuring synaptic content from excitatory and inhibitory synapses using CellProfiler. ***A***, Multiplexed images of PRISM stained DIV21 hippocampal neurons are used as input for the CellProfiler pipeline. Raw images (top) are aligned using MAP2 signal. A white-top filter is applied to enhance puncta (middle). Puncta are thresholded and separated into individual puncta using peak intensity (bottom). ***B***, Synapses are labeled excitatory (E) or inhibitory (I) using the presence or absence of excitatory-specific (vGlut1) or inhibitory-specific (vGAT) markers. Synapses with both markers are labeled as dual (D). Clusters that do not contain either marker are labeled as unknown (U). ***C***, Representative image (top) and enlarged dendrite (bottom) from a MAP2-stained DIV21 hippocampal neuron with excitatory (red) and inhibitory (green) synapses labeled as colored circles. The size of the colored circle represents the relative synapsin1 area. Scale bar: 1 μm.

We designed a Cell Profiler pipeline to automate synapse detection and protein analysis. Analysis of individual synapses from multiplexed images requires three main computational steps: image alignment, synapse segmentation, and synapse alignment. To address these technical requirements, we generated a CellProfiler pipeline that imports multiplexed images (four single-channel images from a single field), aligns images from ten different imaging rounds of four channels using MAP2 staining, and uses the synapsin1 channel for synapse identification. Puncta signals are enhanced using a white top-hat filter and then segmented using intensity peaks (Fig. 1A; Extended Data Fig. 1-1). Following segmentation, each individual punctum from each channel is then assigned to a synapsin1 cluster, depending on the percentage of overlap with the synapsin1 signal (see Materials and Methods). Thus, our “synapses” are a conglomeration of puncta: a single synapsin1 punctum and the collection of individual puncta from the other channels that overlap with that synapsin1 cluster. A large number of geometric and intensity feature calculations are then performed to enable a comprehensive and detailed phenotypic analysis (Extended Data Fig. 1-2). Specifically, we measured multiple intensity and shape features for each punctum to later identify synaptic clusters.

10.1523/ENEURO.0286-20.2020.f1-1 Extended Data Figure 1-1 Overview of CellProfiler workflow. The CellProfiler pipeline takes maximum projection images as input then aligns the images and performs illumination correction by subtracting background signal. The pipeline next defines nuclei objects using DAPI, dendrite objects, using MAP2 and puncta objects using the various PRISM and IF staining. In the last stage of the pipeline, the puncta objects are organized into synapses. Puncta objects are considered synaptic if the objects are outside of the nuclei, are within eight pixels of the dendritic mask and are overlapping with synapsin1 objects. Data are exported to a csv file (green) and multiple images (purple) are created during each stage for quality control. Download Figure 1-1, TIF file.

**Figure 2.**
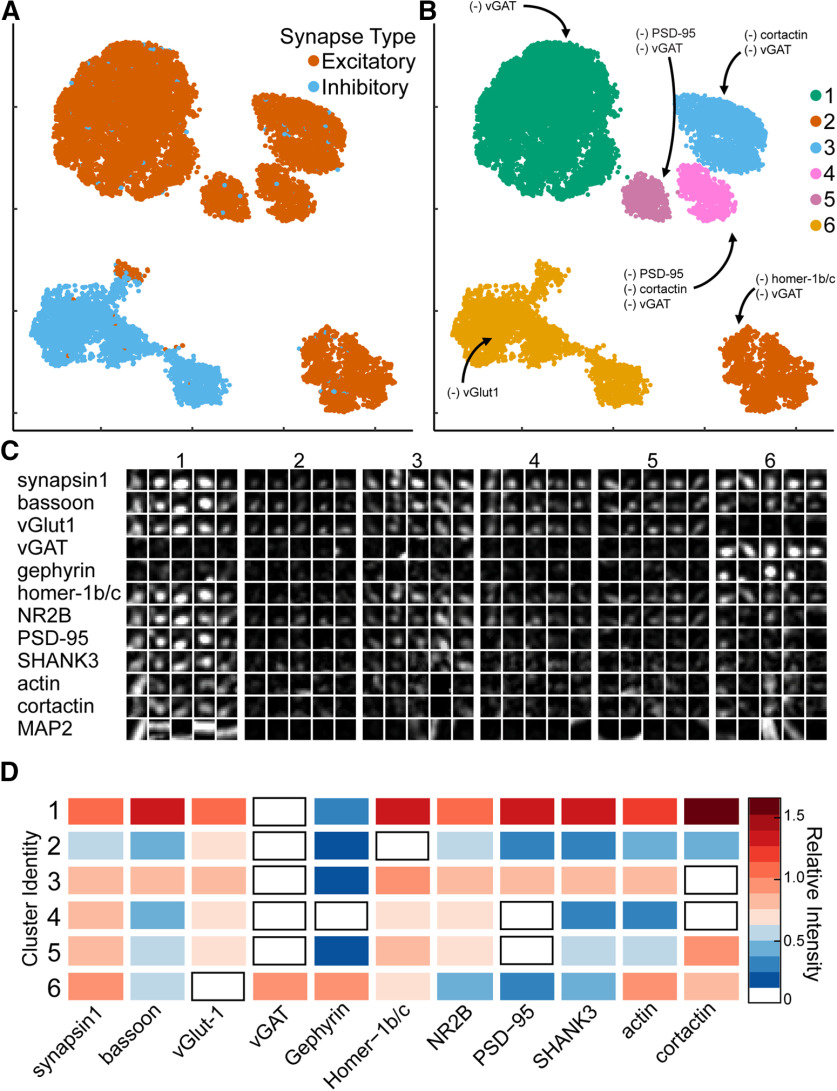
Synaptic cluster identification using UMAP. ***A***, UMAP plots of individual synapses (*n* = 10,000) using CellProfiler output separates excitatory (red) and inhibitory (blue) synapses into two major clusters with multiple subclusters. ***B***, Unique clusters identified by HDBSCAN. (–) indicates synaptic target below limit of detection. ***C***, Representative synapses from each cluster. ***D***, Heatmap indicates the average relative intensities for each synaptic target within each cluster. All values are normalized to untreated mean integrated intensity.

10.1523/ENEURO.0286-20.2020.f1-2 Extended Data Figure 1-2 Synaptic properties measured by CellProfiler. The various intensity, shape, distance and object number measurements performed by CellProfiler. Each measurement is performed on each target from PRISM staining. Download Figure 1-2, PDF file.

Primary rat hippocampal neurons were grown for 19 d in culture, then fixed and stained with our PRISM antibodies/peptides: PSD-95, synapsin1, bassoon, actin, cortactin, homer-1b/c, SHANK3, NR2B, and vGlut1 plus two antibodies against inhibitory synaptic proteins, vGAT and gephyrin (Fig. 1*A*). Synapses were defined as excitatory when the presynaptic proteins synapsin1 and vGlut1 were present versus inhibitory when synapsin1 and vGAT were present (Fig. 1*C*,*D*). Under this classification criterion, 66% of detected puncta were defined as synapses and used for further characterization (Extended Data Fig. 1-3). Synapses that failed to meet our criteria of classification for glutamatergic or GABAergic synapses were excluded from the analysis. These types of puncta were labeled as unclassified (Extended Data Fig. 1-3) because they either lacked vGlut1 or vGAT (∼22%, “unknown”), or contained both synaptic markers (∼12%, “dual”). Thus, the CellProfiler pipeline enabled us to restrict our analysis of synaptic composition to synapses we could confidently identify.

**Figure 3.**
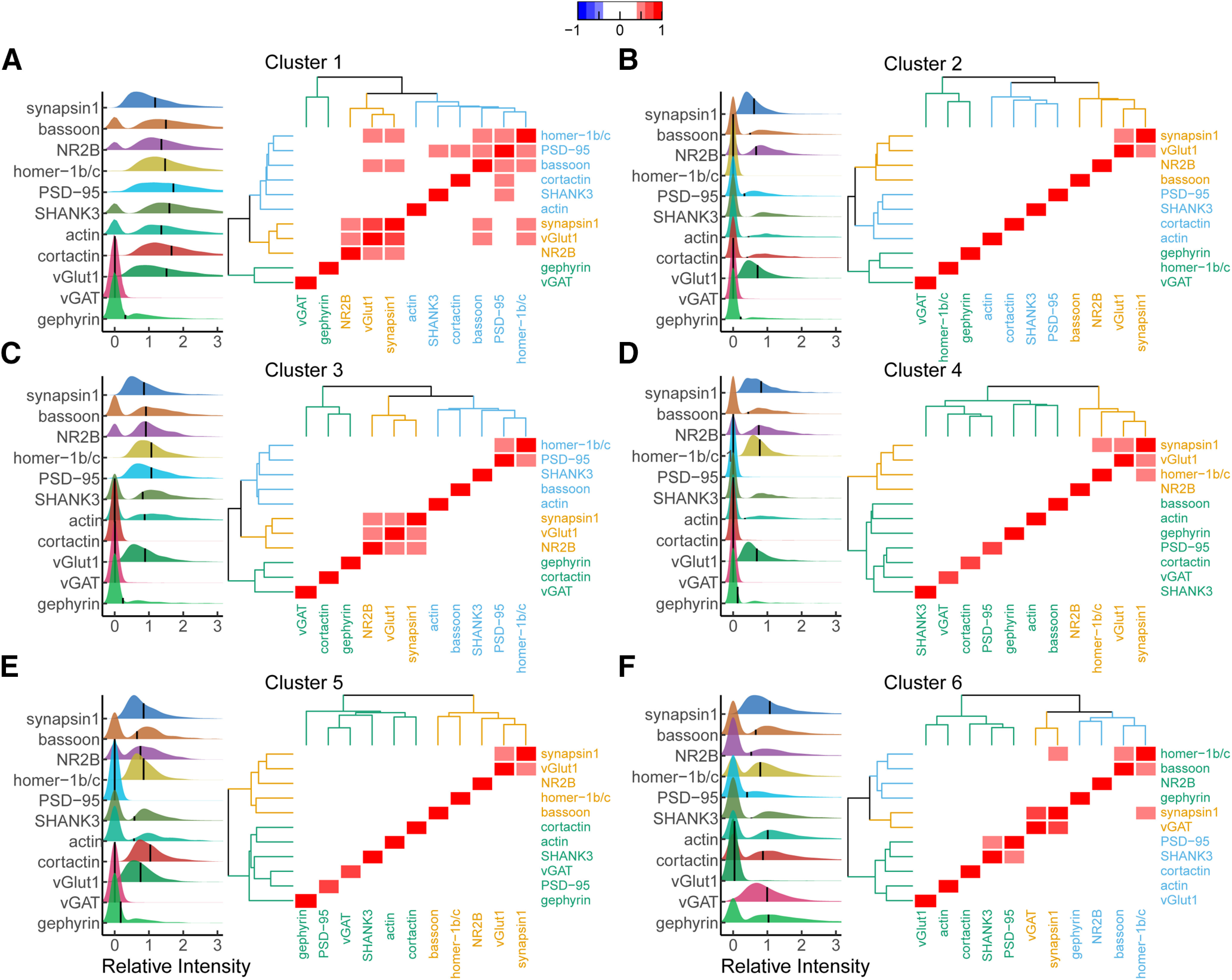
Comparison of synaptic intensity and protein relationships among all proteins within each cluster. Ridgeline plots of relative synaptic intensity for each cluster group (***A***–***F***) using HDBSCAN. Horizontal black line represents cluster mean intensity. All values are normalized to untreated mean integrated intensity. The first peak indicates synapses with integrated intensity of zero. Heatmap indicates the correlation coefficient between each protein. Correlation values between −0.4 and 0.4 obscured to highlight strong correlations. Dendrograms surrounding heatmap show hierarchical clustering of proteins within each cluster.

10.1523/ENEURO.0286-20.2020.f1-3 Extended Data Figure 1-3 Classification of synapses using CellProfiler. Synapses are classified based on the presence of synapsin1, vGlut1, and vGAT. Synapses with synapsin1 and only vGlut1 are classified as excitatory. Synapses with synapsin1 and only vGAT are classified as inhibitory. The remaining synapses are unclassified and contain either synapsin1 alone, or synapsin1 and both vGlut1 and vGAT. Download Figure 1-3, TIF file.

### Characterization of synapse diversity

To characterize synaptic diversity in hippocampal cultured neurons, UMAP was applied to a subset of the CellProfiler pipeline output (Fig. 2*A*). In total, 15 separate intensity measurements, 14 separate punctum shape measurements, quantification of punctum distances to synapsin1, and quantification of the number of puncta associated with each synapse were measured using CellProfiler (Extended Data Fig. 1-2). Feature reduction (see Materials and Methods) was used to isolate the most important features before clustering. The UMAP output shows clearly distinct excitatory and inhibitory synapses, which were found at a ratio of 5 excitatory to 1 inhibitory, consistent with the ratio observed using the CellProfiler pipeline to classify synapses (Fig. 2*A*). Most (98.9%) of the synapses present in the excitatory clusters were positive for vGlut1, staining whereas only 1.1% of the synapses in those clusters contained vGAT. Similarly, 97.3% of synapses present in the inhibitory cluster contained synapsin1 and vGAT and only 2.7% of these synapses contained synapsin1 and vGlut1.

To evaluate the quality of the segmentation, we assessed whether canonical proteins specific to one type of synapse were present at excitatory and inhibitory synapses. We found the majority (78.8%) of the excitatory synapses did not contain the inhibitory target gephyrin and those that did express gephyrin had lower synaptic levels compared with inhibitory synapses (Table 1, a). Moreover, synapsin1 and gephyrin were further apart (mean distance 1.22 ± 0.02 pixels) than synapsin1 and the excitatory marker PSD-95 (0.92 ± 0.01 pixels), suggesting the detected gephyrin was likely outside of these excitatory synapses, but overlapping because of limited spatial resolution. Similarly, the excitatory marker PSD-95 was detectable in 35.7% of inhibitory synapses; however, synaptic levels of PSD-95 within the inhibitory cluster were reduced (Table 1, a) and the mean distance between synapsin1 and PSD-95 (1.5 ± 0.04 pixels) was almost twice that of synapsin1 and gephyrin (0.8 ± 0.02 pixels). Thus, given the limits of the imaging resolution and density of the neuronal culture, the image segmentation may have incorrectly assigned proteins to some synapses; however, the assignment of proteins to synapses can be further refined, as needed, using features such as protein levels and distance to synapsin1.

We examined the UMAP output in further detail to identify and characterize synapses with distinct protein expression profiles. Using HDBSCAN applied to the UMAP output, we identified six unique clusters of synaptic subtypes (Fig. 2*B*,*C*). Subsequently, we generated heatmaps (Fig. 2*D*) and additional scatterplots (Extended Data Figs. 2-1, 2-2, 2-3, 2-4, 2-5, 2-6, 2-7, 2-8, 2-9, 2-10, 2-11, 2-12, 2-13) to distinguish differences among these clusters. Based on the relative vGlut1 and vGAT levels, excitatory synapses correspond to Clusters 1–5 and inhibitory synapses are contained in Cluster 6. Cluster 1 was the largest cluster with 57.9% of the excitatory synapses and had the highest levels of synaptic proteins for all targets relative to the other excitatory clusters (Fig. 2*D*; Extended Data Fig. 2-14). Compared with Cluster 1, Clusters 2–5 contain lower levels of all synaptic proteins and each of these clusters had very low levels of one or more postsynaptic scaffolding or cytoskeletal protein. To investigate in more detail the differences among the excitatory synaptic clusters and identify the distinguishing features for each synapse subtype, we measured the protein intensity profiles, correlation coefficients, and performed hierarchical clustering for each of the individual clusters (Fig. 3).

10.1523/ENEURO.0286-20.2020.f2-1 Extended Data Figure 2-1 UMAP analysis of synapsin1 shape measurements. UMAP analysis of synapses colored for indicated synapsin1 shape metrics. Download Figure 2-1, TIF file.

10.1523/ENEURO.0286-20.2020.f2-2 Extended Data Figure 2-2 UMAP analysis of puncta number per synapse. UMAP analysis shows synapses colored for the number of puncta per synapse for each synaptic target. Download Figure 2-2, TIF file.

10.1523/ENEURO.0286-20.2020.f2-3 Extended Data Figure 2-3 UMAP analysis of relative integrated intensity. UMAP analysis of synapses colored for relative integrated intensity of each synaptic target. Scale bar shows log values. Download Figure 2-3, TIF file.

**Figure 4.**
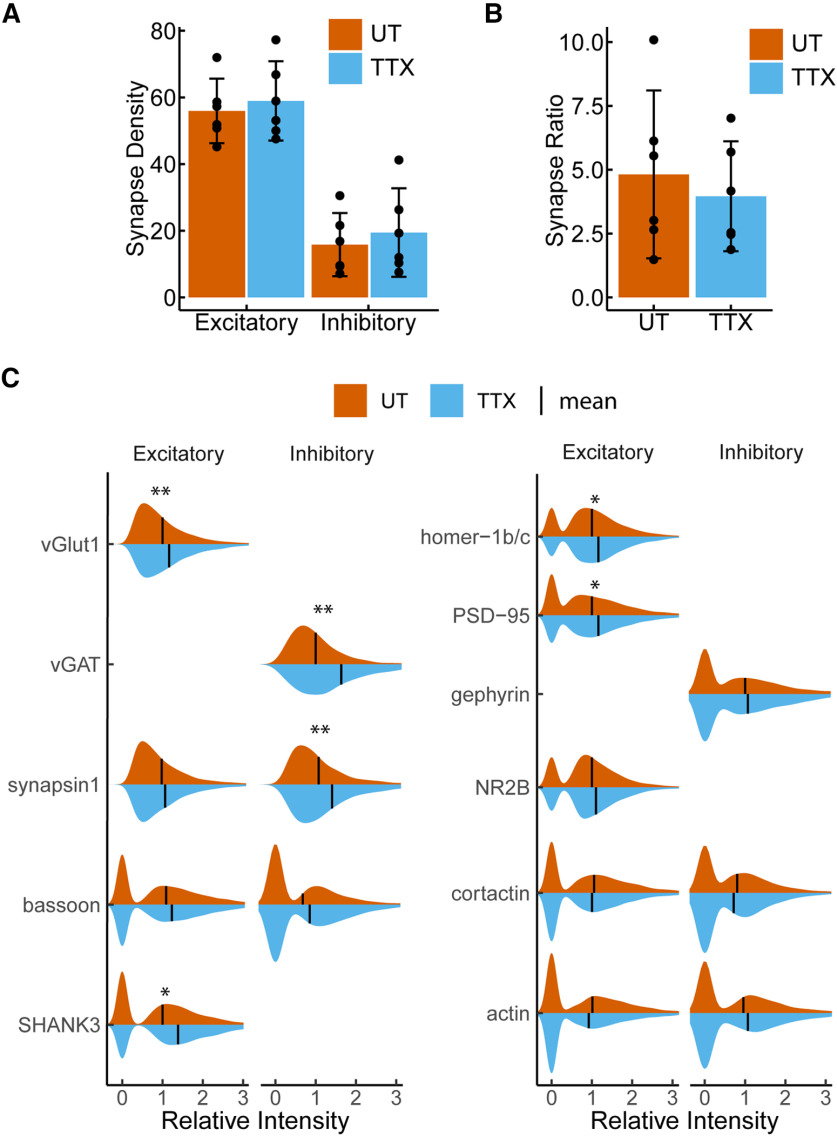
Excitatory and inhibitory synaptic density does not change in response to 48-h TTX treatment. ***A***, Quantification of excitatory and inhibitory synaptic density from untreated (red) and TTX-treated (blue) cells. Bar height represents the mean number of synapses per 100-μm length of dendrite. ***B***, Quantification of the excitatory:inhibitory synaptic ratio in untreated (red) and TTX-treated (blue) neurons. Bar height represents mean excitatory:inhibitory ratio. Error bars indicate 95% confidence intervals. Closed circles indicate results from individual replicates *n* = 6. ***C***, Violin plots of relative synaptic intensity for synaptic targets at excitatory synapses (left) and inhibitory synapses (right) from untreated (red) and TTX-treated cells (blue). Black line indicates the mean intensity; *p* values are computed using Student’s *t* test on the mean Integrated Intensity with *n* = 6 (UT) and *n* = 5 (TTX) replicates. All values are normalized to untreated mean integrated intensity; **p <* *0.01, *p <* *0.05.

10.1523/ENEURO.0286-20.2020.f2-4 Extended Data Figure 2-4 UMAP analysis of relative integrated intensity of puncta edge. UMAP analysis of synapses colored for relative integrated intensity of the edge of each punctum for each synaptic target. Scale bar show log values. Download Figure 2-4, TIF file.

**Figure 5.**
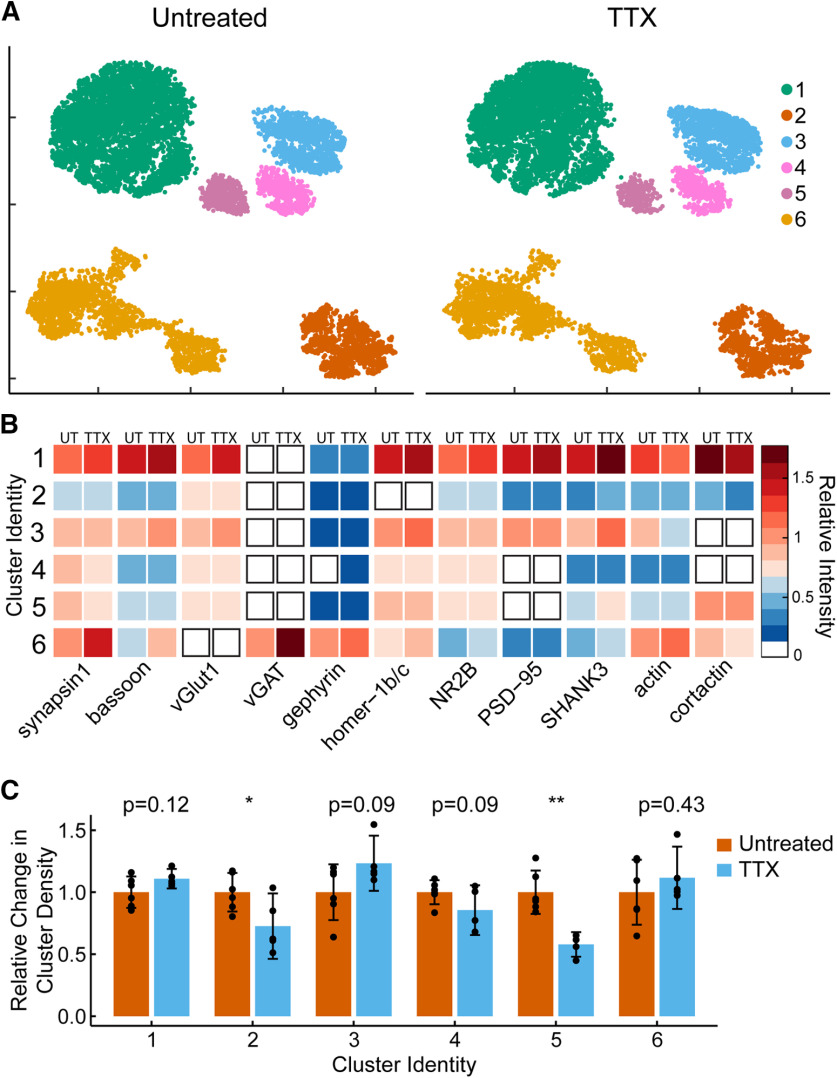
Characterizing changes within clusters in response to TTX treatment. ***A***, UMAP plots of individual synapses (*n* = 10,000) separated by treatment group. ***B***, Heatmap indicates the average relative intensities for each synaptic target within each cluster. Left side of the column is the untreated sample, the right side of the column is the TTX-treated sample. All values are normalized to untreated mean integrated intensity. ***C***, Bar heights show synapse density, relative to untreated groups, within each HDBSCAN identified cluster following TTX treatment; *p* values are computed using Student’s *t* test with *n* = 6 (UT) and *n* = 5 (TTX) replicates; **p <* *0.01, *p <* *0.05. Error bars indicate 95% confidence intervals.

10.1523/ENEURO.0286-20.2020.f2-5 Extended Data Figure 2-5 UMAP analysis of lower quartile intensity. UMAP analysis of synapses colored for the lower quartile intensity (log values) of each punctum for each synaptic target. Download Figure 2-5, TIF file.

10.1523/ENEURO.0286-20.2020.f2-6 Extended Data Figure 2-6 UMAP analysis of median absolute deviation. UMAP analysis of synapses colored for median absolute deviation intensity (log values) of each punctum for each synaptic target. Download Figure 2-6, TIF file.

10.1523/ENEURO.0286-20.2020.f2-7 Extended Data Figure 2-7 UMAP analysis of puncta mass displacement. UMAP analysis of synapses colored for the mass displacement (log values) of each punctum for each synaptic target. Download Figure 2-7, TIF file.

10.1523/ENEURO.0286-20.2020.f2-8 Extended Data Figure 2-8 UMAP analysis of minimum pixel intensity of puncta edge. UMAP analysis of synapses colored for minimum pixel intensity on the edge of each punctum for each synaptic target. Download Figure 2-8, TIF file.

10.1523/ENEURO.0286-20.2020.f2-9 Extended Data Figure 2-9 UMAP analysis of puncta maximum intensity. UMAP analysis of synapses colored for maximum intensity (log values) of each punctum for each synaptic target. Download Figure 2-9, TIF file.

10.1523/ENEURO.0286-20.2020.f2-10 Extended Data Figure 2-10 UMAP analysis of puncta edge standard deviation. UMAP analysis of synapses colored for standard deviation of the edge (log values) of each punctum for each synaptic target. Download Figure 2-10, TIF file.

10.1523/ENEURO.0286-20.2020.f2-11 Extended Data Figure 2-11 UMAP analysis of puncta upper quartile intensity. UMAP analysis of synapses colored for the upper quartile intensity of each punctum for each synaptic target. Download Figure 2-11, TIF file.

10.1523/ENEURO.0286-20.2020.f2-12 Extended Data Figure 2-12 UMAP analysis of distance to synapsin1 puncta centroid. UMAP analysis of synapses colored for distance in pixels (log value) between puncta centroids (synapsin1 and each indicated target). Download Figure 2-12, TIF file.

10.1523/ENEURO.0286-20.2020.f2-13 Extended Data Figure 2-13 UMAP analysis of minimum distance to synapsin1 puncta. UMAP analysis of synapses colored for minimum distance in pixels (log values) between the indicated target and synapsin1. Download Figure 2-13, TIF file.

10.1523/ENEURO.0286-20.2020.f2-14 Extended Data Figure 2-14 Relative synaptic intensities of all targets for each synaptic subtype. Ridgeline plots of relative synaptic intensities for synaptic targets within cluster groups identified using HDBScan. Horizontal black line represents cluster mean intensity. All values are normalized to untreated mean integrated intensity. Percent of synapses with integrated intensity >0 for each cluster. Download Figure 2-14, TIF file.

10.1523/ENEURO.0286-20.2020.f5-1 Extended Data Figure 5-1 Synaptic cluster identification for individual replicates. UMAP plots of individual synapses using CellProfiler output separates excitatory and inhibitory synapses into approximately six unique clusters identified by HDBSCAN. Each cluster is present in each sample indicating unique cluster formation is not an artifact of individual differences in culture conditions across different samples. Download Figure 5-1, TIF file.

Ridgeline plots (Fig. 3, left subpanel) were created for each protein within each cluster to compare the relative synaptic levels of each target and to examine the proportion of synapses with detectable (>0) levels of each target. Additionally, pairwise correlation analysis of the synaptic protein levels was performed to identify proteins with coordinated synaptic expression (Fig. 3, right subpanel). Hierarchical clustering was then performed on the correlation matrix to identify which groups of proteins had similar levels of coordinated synaptic expression. Comparing these features across the excitatory clusters shows that Cluster 1 had the highest level of synaptic proteins, the lowest proportion of synapses with undetectable levels of synaptic proteins (Fig. 3*A–E*, left subpanel), and the most proteins with coordinated synaptic expression (Fig. 3*A–E*, right subpanel) of the excitatory synaptic clusters. Cluster three was the most similar to Cluster 1 as it had the next highest synaptic levels of proteins and the next highest number of proteins with coordinated synaptic expression. Clusters 2, 4, and 5 had lower levels of proteins at synapses compared with Clusters 1 and 3, more synapses with undetectable levels of proteins, and very few proteins with coordinated synaptic expression. In these clusters the proteins with most of the coordinated synaptic expression were synapsin1 and vGlut1. Taken together, these clusters may be synapses in various stages of maturation, synapses actively remodeling or they could represent false synapses caused from staining artifacts.

Cluster 6 contains inhibitory synapses with vGAT detectable in 97.8% of these synapses. Gephyrin levels were also the highest within this cluster relative to all other clusters, but, interestingly, were only detectable in 61% of these synapses. The excitatory postsynaptic scaffolds SHANK3 and PSD-95 were assigned to inhibitory synapses in ∼41% and 36% of the synapses in this cluster. Additionally, homer-1b/c was also found near inhibitory synapses in 73% of these synapses but with lower intensity with respect to Cluster 1 (Fig. 3; Extended Data Fig. 2-14; Table 4, a). Correlation analysis of proteins within this cluster revealed coordinated synaptic expression between vGAT and synapsin1, between PSD-95 and SHANK3, between homer-1b/c and bassoon and surprisingly between synapsin1 and homer-1b/c. Taken together, within this cluster, we only see coordinate synaptic expression among the presynaptic inhibitory synaptic proteins. Additionally, our results suggest homer-1b/c may serve some yet unknown function at inhibitory synapses.

**Table 4:** Statistics table

								Mean			Effect (Cohen’s *d*)					*N*	
	Figure	Target	Group comparison	Data structure	Type of test	Shuffles	*p* value	Difference	95% CI lower	95% CI upper	Effect	95% CI lower	95% CI upper	Power	*p *<* *0.05	UT	TTX
a		Gephyrin distance to synapsin1	Gephyrin positive synapses (UMAP 6 vs UMAP1-5)	Normal	Permutation *t* test	5000	0.002	−0.44	−0.46	−0.40	−16.60	−20.65	−12.49	1.0	*	6	5
	3*A*,*F*	Gephyrin integrated intensity	Gephyrin positive synapses (UMAP 6 vs UMAP1-5)	Normal	Permutation *t* test	5000	0.002	0.35	0.20	0.49	2.70	1.25	5.46	1.0	*	6	5
		PSD-95 distance to synapsin1	PSD-95 positive synapses (UMAP6 vs UMAP1-5)	Normal	Permutation *t* test	5000	0.003	−0.56	−0.70	−0.47	−5.67	−7.51	−4.11	1.0	*	6	5
	3*A*,*F*	PSD-95 integrated intensity	PSD-95 positive synapses (UMAP6 vs UMAP1-5)	Normal	Permutation *t* test	5000	0.007	−0.23	−0.31	−0.10	−2.56	−6.14	−0.69	1.0	*	6	5
	3*A*,*F*	Homer1-b/c integrated intensity	Homer1-b/c positive synapses (UMAP6 vs UMAP1)	Normal	Permutation *t* test	5000	0.003	−0.56	−0.67	−0.43	−5.08	−7.32	−3.58	1.0	*	6	5
b	4*A*	Excitatory synapse number	Untreated vs TTX	Normal	Permutation *t* test	5000	0.626	2.99	−7.40	15.23	0.29	−1.10	1.59	0.1		6	6
		Inhibitory synapse number	Untreated vs TTX	Normal	Permutation *t* test	5000	0.592	3.62	−7.56	16.24	0.33	−1.03	1.69	0.1		6	6
	4*B*	Excitatory:inhibitory synapse ratio	Untreated vs TTX	Normal	Permutation *t* test	5000	0.592	−0.86	−4.03	1.78	−0.32	−1.64	1.13	0.1		6	6
c	4*C*	vGlut1 integrated intensity	Untreated vs TTX (excitatory synapses)	Normal	Permutation *t* test	5000	0.006	0.17	0.10	0.24	2.82	1.17	5.03	1.0	*	6	5
		vGAT integrated intensity	Untreated vs TTX (inhibitory synapses)	Normal	Permutation *t* test	5000	0.001	0.60	0.44	0.75	4.31	2.84	6.50	1.0	*	6	5
		Synapsin1 integrated intensity	Untreated vs TTX (excitatory synapses)	Normal	Permutation *t* test	5000	0.110	0.10	−0.02	0.18	1.11	−0.52	4.00	0.4		6	5
		Synapsin1 integrated intensity	Untreated vs TTX (inhibitory synapses)	Normal	Permutation *t* test	5000	0.004	0.35	0.23	0.47	3.11	1.53	4.95	1.0	*	6	5
		Bassoon integrated intensity	Untreated vs TTX (excitatory synapses)	Normal	Permutation *t* test	5000	0.129	0.18	−0.04	0.38	1.02	−0.66	2.49	0.3		6	5
		Bassoon integrated intensity	Untreated vs TTX (inhibitory synapses)	Normal	Permutation *t* test	5000	0.360	0.11	−0.12	0.32	0.57	−0.98	2.07	0.1		6	5
		SHANK3 integrated intensity	Untreated vs TTX (excitatory synapses)	Normal	Permutation *t* test	5000	0.008	0.35	0.12	0.46	2.45	0.60	7.29	0.9	*	6	5
		Homer1-b/c integrated intensity	Untreated vs TTX (excitatory synapses)	Normal	Permutation *t* test	5000	0.037	0.15	0.02	0.26	1.54	−0.02	3.44	0.6	*	6	5
		PSD-95 integrated intensity	Untreated vs TTX (excitatory synapses)	Normal	Permutation *t* test	5000	0.017	0.15	0.06	0.24	1.93	0.71	3.31	0.8	*	6	5
		Gephyrin integrated intensity	Untreated vs TTX (inhibitory synapses)	Normal	Permutation *t* test	5000	0.610	0.07	−0.14	0.35	0.31	−1.24	1.72	0.1		6	5
		NR2B integrated intensity	Untreated vs TTX (excitatory synapses)	Normal	Permutation *t* test	5000	0.089	0.12	−0.02	0.22	1.18	−0.48	3.26	0.4		6	5
		Cortactin integrated intensity	Untreated vs TTX (excitatory synapses)	Normal	Permutation *t* test	5000	0.549	−0.09	−0.33	0.18	−0.39	−2.45	1.07	0.1		6	5
		Cortactin integrated intensity	Untreated vs TTX (inhibitory synapses)	Normal	Permutation *t* test	5000	0.589	−0.09	−0.41	0.24	−0.31	−1.87	1.14	0.1		6	5
		Actin integrated intensity	Untreated vs TTX (excitatory synapses)	Normal	Permutation *t* test	5000	0.475	−0.07	−0.26	0.04	−0.53	−1.80	1.06	0.1		6	5
		Actin integrated intensity	Untreated vs TTX (inhibitory synapses)	Normal	Permutation *t* test	5000	0.794	0.04	−0.22	0.38	0.17	−1.48	2.06	0.1		6	5
d	5*C*	Synapsin1 UMAP1	Untreated vs TTX	Normal	Permutation *t* test	5000	0.202	0.13	−0.05	0.30	0.84	−0.72	2.49	0.2		5	
		Synapsin1 UMAP2	Untreated vs TTX	Normal	Permutation *t* test	5000	0.479	−0.03	−0.12	0.03	−0.47	−1.83	1.09	0.1		6	5
		Synapsin1 UMAP3	Untreated vs TTX	Normal	Permutation *t* test	5000	0.569	0.03	−0.10	0.09	0.36	−1.23	3.70	0.1		6	5
		Synapsin1 UMAP4	Untreated vs TTX	Normal	Permutation *t* test	5000	0.237	−0.06	−0.17	0.03	−0.82	−2.54	1.03	0.2		6	5
		Synapsin1 UMAP5	Untreated vs TTX	Normal	Permutation *t* test	5000	0.099	−0.09	−0.18	0.01	−1.09	−2.63	0.48	0.4		6	5
		Synapsin1 UMAP6	Untreated vs TTX	Normal	Permutation *t* test	5000	0.005	0.37	0.24	0.49	3.09	1.64	4.75	1.0	*	6	5
		Gephyrin UMAP1	Untreated vs TTX	Normal	Permutation *t* test	5000	0.464	0.02	−0.03	0.07	0.47	−0.92	1.96	0.1		6	5
		Gephyrin UMAP2	Untreated vs TTX	Normal	Permutation *t* test	5000	0.261	−0.03	−0.08	0.02	−0.68	−2.13	0.68	0.2		6	5
		Gephyrin UMAP3	Untreated vs TTX	Normal	Permutation *t* test	5000	0.345	−0.03	−0.08	0.02	−0.61	−1.96	0.76	0.1		6	5
		Gephyrin UMAP4	Untreated vs TTX	Normal	Permutation *t* test	5000	0.304	0.02	−0.02	0.06	0.69	−1.58	2.43	0.2		6	5
		Gephyrin UMAP5	Untreated vs TTX	Normal	Permutation *t* test	5000	0.647	0.02	−0.05	0.08	0.30	−1.82	2.69	0.1		6	5
		Gephyrin UMAP6	Untreated vs TTX	Normal	Permutation *t* test	5000	0.733	0.03	−0.12	0.25	0.20	−1.41	1.56	0.1		6	5
		Homer1-b/c UMAP1	Untreated vs TTX	Normal	Permutation *t* test	5000	0.221	0.09	−0.05	0.23	0.81	−0.89	2.60	0.2		6	5
		Homer1-b/c UMAP1	Untreated vs TTX	Normal	Permutation *t* test	5000	0.452	0.00	0.00	0.00	−0.81	−1.64	−0.40	0.2		6	5
		Homer1-b/c UMAP1	Untreated vs TTX	Normal	Permutation *t* test	5000	0.438	0.05	−0.07	0.16	0.49	−1.17	2.16	0.1		6	5
		Homer1-b/c UMAP1	Untreated vs TTX	Normal	Permutation *t* test	5000	0.660	0.01	−0.04	0.08	0.29	−1.70	2.07	0.1		6	5
		Homer1-b/c UMAP1	Untreated vs TTX	Normal	Permutation *t* test	5000	0.474	0.02	−0.05	0.08	0.41	−1.23	2.01	0.1		6	5
		Homer1-b/c UMAP1	Untreated vs TTX	Normal	Permutation *t* test	5000	0.031	0.19	0.07	0.29	1.80	0.48	4.20	0.8	*	6	5
		NR2B UMAP1	Untreated vs TTX	Normal	Permutation *t* test	5000	0.144	0.10	−0.03	0.22	0.97	−0.48	2.77	0.3		6	5
		NR2B UMAP2	Untreated vs TTX	Normal	Permutation *t* test	5000	0.827	−0.02	−0.14	0.11	−0.15	−1.71	1.68	0.1		6	5
		NR2B UMAP3	Untreated vs TTX	Normal	Permutation *t* test	5000	0.312	0.07	−0.04	0.17	0.63	−0.95	2.41	0.2		6	5
		NR2B UMAP4	Untreated vs TTX	Normal	Permutation *t* test	5000	0.661	0.05	−0.18	0.28	0.26	−1.50	2.35	0.1		6	5
		NR2B UMAP5	Untreated vs TTX	Normal	Permutation *t* test	5000	0.744	−0.03	−0.20	0.12	−0.20	−1.80	1.34	0.1		6	5
		NR2B UMAP6	Untreated vs TTX	Normal	Permutation *t* test	5000	0.533	0.14	−0.33	0.54	0.37	−1.08	1.97	0.1		6	5
		PSD-95 UMAP1	Untreated vs TTX	Normal	Permutation *t* test	5000	0.313	0.06	−0.04	0.17	0.65	−0.85	2.04	0.2		6	5
		PSD-95 UMAP2	Untreated vs TTX	Normal	Permutation *t* test	5000	0.053	0.06	0.01	0.11	1.43	−0.33	3.18	0.6		6	5
		PSD-95 UMAP3	Untreated vs TTX	Normal	Permutation *t* test	5000	0.778	0.02	−0.10	0.15	0.17	−1.28	1.56	0.1		6	5
		PSD-95 UMAP4	Untreated vs TTX	Normal	Permutation *t* test	5000	0.882	0.00	0.00	0.00	−0.12	−1.47	1.58	0.1		6	5
		PSD-95 UMAP5	Untreated vs TTX	Normal	Permutation *t* test	5000	0.184	0.00	0.00	0.01	0.86	0.40	1.50	0.2		6	5
		PSD-95 UMAP6	Untreated vs TTX	Normal	Permutation *t* test	5000	0.729	−0.01	−0.08	0.07	−0.21	−1.69	1.26	0.1		6	5
		SHANK3 UMAP1	Untreated vs TTX	Normal	Permutation *t* test	5000	0.018	0.23	0.11	0.35	2.07	0.81	4.11	0.9	*	6	5
		SHANK3 UMAP2	Untreated vs TTX	Normal	Permutation *t* test	5000	0.011	0.10	0.04	0.15	1.94	0.55	3.24	0.8	*	6	5
		SHANK3 UMAP3	Untreated vs TTX	Normal	Permutation *t* test	5000	0.019	0.20	0.08	0.30	1.85	0.55	4.35	0.8	*	6	5
		SHANK3 UMAP4	Untreated vs TTX	Normal	Permutation *t* test	5000	0.186	0.06	−0.03	0.14	0.85	−0.62	2.44	0.2		6	5
		SHANK3 UMAP5	Untreated vs TTX	Normal	Permutation *t* test	5000	0.285	0.06	−0.04	0.16	0.67	−0.80	2.17	0.2		6	5
		SHANK3 UMAP6	Untreated vs TTX	Normal	Permutation *t* test	5000	0.360	0.07	−0.06	0.22	0.58	−0.84	2.15	0.1		6	5
		Actin UMAP1	Untreated vs TTX	Normal	Permutation *t* test	5000	0.345	−0.10	−0.28	0.06	−0.60	−1.95	1.11	0.1		6	5
		Actin UMAP2	Untreated vs TTX	Normal	Permutation *t* test	5000	0.927	0.00	−0.05	0.06	−0.06	−1.54	1.59	0.1		6	5
		Actin UMAP3	Untreated vs TTX	Normal	Permutation *t* test	5000	0.407	−0.06	−0.18	0.06	−0.56	−2.01	1.13	0.1		6	5
		Actin UMAP4	Untreated vs TTX	Normal	Permutation *t* test	5000	0.721	−0.01	−0.07	0.05	−0.21	−1.70	1.26	0.1		6	5
		Actin UMAP5	Untreated vs TTX	Normal	Permutation *t* test	5000	0.923	−0.01	−0.18	0.07	−0.11	−1.72	2.14	0.1		6	5
		Actin UMAP6	Untreated vs TTX	Normal	Permutation *t* test	5000	0.876	0.01	−0.14	0.19	0.10	−1.31	1.65	0.1		6	5
		Bassoon UMAP1	Untreated vs TTX	Normal	Permutation *t* test	5000	0.250	0.08	−0.04	0.21	0.74	−0.90	1.94	0.2		6	5
		Bassoon UMAP2	Untreated vs TTX	Normal	Permutation *t* test	5000	0.593	−0.02	−0.11	0.06	−0.33	−1.97	1.56	0.1		6	5
		Bassoon UMAP3	Untreated vs TTX	Normal	Permutation *t* test	5000	0.064	0.09	0.02	0.22	1.16	−0.33	2.12	0.4		6	5
		Bassoon UMAP4	Untreated vs TTX	Normal	Permutation *t* test	5000	0.877	0.01	−0.09	0.13	0.09	−2.47	3.45	0.1		6	5
		Bassoon UMAP5	Untreated vs TTX	Normal	Permutation *t* test	5000	0.569	0.02	−0.05	0.08	0.33	−1.26	2.33	0.1		6	5
		Bassoon UMAP6	Untreated vs TTX	Normal	Permutation *t* test	5000	0.281	0.07	−0.06	0.19	0.64	−0.83	2.20	0.2		6	5
		Cortactin UMAP1	Untreated vs TTX	Normal	Permutation *t* test	5000	0.201	−0.09	−0.24	0.01	−0.84	−2.03	0.63	0.2		6	5
		Cortactin UMAP2	Untreated vs TTX	Normal	Permutation *t* test	5000	0.573	−0.02	−0.11	0.05	−0.34	−1.81	1.07	0.1		6	5
		Cortactin UMAP3	Untreated vs TTX	Normal	Permutation *t* test	5000	0.390	0.00	0.00	0.00	0.55	−0.98	2.37	0.1		6	5
		Cortactin UMAP4	Untreated vs TTX	Normal	Permutation *t* test	5000	0.867	0.00	0.00	0.00	0.11	−1.21	1.57	0.1		6	5
		Cortactin UMAP5	Untreated vs TTX	Normal	Permutation *t* test	5000	0.781	−0.02	−0.12	0.11	−0.16	−2.09	1.78	0.1		6	5
		Cortactin UMAP6	Untreated vs TTX	Normal	Permutation *t* test	5000	0.585	−0.06	−0.24	0.14	−0.33	−1.87	1.10	0.1		6	5
		vGlut1 UMAP1	Untreated vs TTX	Normal	Permutation *t* test	5000	0.044	0.19	0.01	0.30	1.49	−0.25	3.75	0.6	*	6	5
		vGlut1 UMAP2	Untreated vs TTX	Normal	Permutation *t* test	5000	0.252	0.03	−0.02	0.08	0.78	−0.83	2.69	0.2		6	5
		vGlut1 UMAP3	Untreated vs TTX	Normal	Permutation *t* test	5000	0.045	0.08	0.01	0.16	1.38	−0.39	3.00	0.5	*	6	5
		vGlut1 UMAP4	Untreated vs TTX	Normal	Permutation *t* test	5000	0.231	0.03	−0.02	0.07	0.77	−0.83	2.44	0.2		6	5
		vGlut1 UMAP5	Untreated vs TTX	Normal	Permutation *t* test	5000	0.722	0.01	−0.05	0.09	0.21	−2.28	3.33	0.1		6	5
		vGlut1 UMAP6	Untreated vs TTX	Normal	Permutation *t* test	5000	0.358	−0.01	−0.02	0.01	−0.57	−2.24	1.05	0.1		6	5
		vGAT UMAP1	Untreated vs TTX	Normal	Permutation *t* test	5000	0.386	0.00	−0.01	0.00	−0.60	−1.69	1.01	0.1		6	5
		vGAT UMAP2	Untreated vs TTX	Normal	Permutation *t* test	5000	0.883	0.00	0.00	0.00	0.08	−1.41	1.55	0.1		6	5
		vGAT UMAP3	Untreated vs TTX	Normal	Permutation *t* test	5000	0.101	−0.01	−0.02	0.00	−1.03	−1.98	0.55	0.3		6	5
		vGAT UMAP4	Untreated vs TTX	Normal	Permutation *t* test	5000	0.861	0.00	0.00	0.01	0.06	−1.31	1.57	0.1		6	5
		vGAT UMAP5	Untreated vs TTX	Normal	Permutation *t* test	5000	0.101	−0.01	−0.01	0.00	−1.13	−2.16	−0.49	0.4		6	5
		vGAT UMAP6	Untreated vs TTX	Normal	Permutation *t* test	5000	0.002	0.86	0.61	1.08	3.99	2.55	6.30	1.0	*	6	5
e	5*C*	UMAP1 synapse number	Untreated vs TTX	Normal	Permutation *t* test	5000	0.117	0.11	0.01	0.22	1.09	−0.56	2.56	0.4		6	5
		UMAP2 synapse number	Untreated vs TTX	Normal	Permutation *t* test	5000	0.042	−0.27	−0.47	−0.04	−1.52	−3.59	0.15	0.6	*	6	5
		UMAP3 synapse number	Untreated vs TTX	Normal	Permutation *t* test	5000	0.092	0.23	0.07	0.53	1.17	0.10	2.19	0.4		6	5
		UMAP4 synapse number	Untreated vs TTX	Normal	Permutation *t* test	5000	0.091	−0.14	−0.28	0.03	−1.13	−3.51	0.56	0.4		6	5
		UMAP5 synapse number	Untreated vs TTX	Normal	Permutation *t* test	5000	0.002	−0.42	−0.60	−0.30	−3.12	−4.32	−1.96	1.0	*	6	5
		UMAP6 synapse number	Untreated vs TTX	Normal	Permutation *t* test	5000	0.428	0.12	−0.11	0.42	0.51	−0.84	1.85	0.1		6	5

Two-tailed permutation *t* tests, mean confidence intervals, and effect size were calculated the from 5000 reshuffles of the untreated (*n* = 6) and TTX-treated (*n* = 5) samples. The *p* values represent the likelihood of observing the reported effect size, if the null hypothesis of zero difference is true. Power analysis represents the probability of observing effect size if the null hypothesis of zero difference is true.

### Response of excitatory and inhibitory synapses to activity blockade

The phenomena of neurons eliciting compensatory structural changes within synapses to maintain network homeostatic balance in response to chronic activity blockade has been characterized in numerous studies (Turrigiano, 2008). However, the overview of simultaneous changes in inhibitory and excitatory synapses has been limited by conventional immunocytochemistry. We used PRISM staining to simultaneously investigate the effect of chronic activity blockade on excitatory and inhibitory synapses. For this, the distribution pattern of excitatory and inhibitory synapses was determined by using the classification method established above. We found a higher abundance of excitatory inputs compared with inhibitory on cultured neurons (Fig. 4*B*). Under control conditions, glutamatergic synapses have a density of 56 ± 9.6 synapses per 100-μm length of dendrite, whereas GABAergic synapses density was 15.8 ± 9.5 per 100 μm, which gives an average excitatory to inhibitory synapses ratio of 5 ± 3:1 calculated per replicate (Fig. 4*B*; Table 4, b). Accordingly with the well-established model that neurons coordinate their excitatory and inhibitory inputs to establish and maintain a constant excitatory-to-inhibitory (E/I) ratio which is essential for circuit function and stability (Zhou et al., 2014; He et al., 2018; He and Cline, 2019), we observed that TTX treatment did not alter this ratio or density of synapses in hippocampal cultured neurons (Fig. 4*A*,*B*; Table 4, b).

It is well known that homeostatic plasticity regulates the relative strength of excitatory and inhibitory synapses to keep relatively stable firing rates of neurons by increasing synaptic levels of proteins that regulate excitatory signaling (Turrigiano et al., 1998; Turrigiano and Nelson, 2000, 2004; Liu, 2004; Hartman et al., 2006; Turrigiano, 2008, 2012). We applied our imaging platform and CellProfiler software to identify excitatory and inhibitory synapses and to examine the response of 11 synaptic targets simultaneously following induction of homeostatic synaptic plasticity. Interestingly, using relative intensity measurements to evaluate protein abundance, we observed different patterns of response to neuronal activity blockade depending whether proteins are present at excitatory or inhibitory synapses or are presynaptic or postsynaptic (Fig. 4*C*). The neurotransmitter transporters at presynaptic excitatory and inhibitory terminals, vGlut1 and vGAT, respectively, both show a significant increase in response to TTX treatment. In contrast, synapsin1, a protein associated with the reserve pool of synaptic vesicles, shows increased levels in response to TTX treatment exclusively at inhibitory synapses. Additionally, at both excitatory and inhibitory synapses there was no change in the levels of the presynaptic protein bassoon. However, overall bassoon levels were lower at inhibitory synapses compared with excitatory synapses. On the postsynaptic side, the levels of the excitatory scaffolding proteins SHANK3, homer-1b/c, and PSD-95 increased in the presence of TTX, while no changes in the inhibitory postsynaptic scaffold gephyrin were observed at inhibitory synapses. Interestingly, no changes were observed in the cytoskeletal proteins cortactin and actin at excitatory or inhibitory synapses (Fig. 4*C*; Table 4, c). Thus, we observe that excitatory synapses respond both postsynaptically and presynaptically to neuronal activity suppression while at inhibitory synapses the response is mainly presynaptic.

### Alterations in excitatory and inhibitory synapses within synaptic subgroups following activity blockade

We further assessed the effect of activity blockage via TTX on synaptic subtypes and found that the same clusters described in control neurons are present after chronic neuronal activity blockage (Fig. 5). Analysis of synaptic intensity revealed no differences for most synaptic targets within each cluster (Fig. 5*B*; Table 4, d). Instead, we found that following 48-h TTX treatment, the number of synapses present in each cluster changed. Specifically, there was a small decrease in the number of synapses within Clusters 2, 4, and 5 (*p *<* *0.05, *p *=* *0.09, and *p *<* *0.001; Fig. 5*C*; Table 4, e). Taken together, the results indicate that TTX treatment does not produce unique synaptic subtypes (based on the proteins we examined), but it does change the number of synapses present within each cluster, specifically, reducing the number of synapses in clusters defined by low protein expression.

## Discussion

We simultaneously characterized inhibitory and excitatory synaptic content from multiplexed imaging using PRISM and CellProfiler, offering detailed molecular insight into synapse complexity at the single-synapse level, including excitatory and inhibitory components.

Synapse identification using synapsin1 in combination with either vGlut1 or vGAT indicated an E/I ratio of ∼5 ± 3:1 in hippocampal neurons. In cultured neurons, the E/I ratio reported using imaging techniques is variable and dependent on multiple factors such as brain region and the age of the culture. In hippocampal culture, E/I ratios range between 2.5:1 and 17:1 (Gulyás et al., 1999; Megías et al., 2001; Liu, 2004; Harrill et al., 2015), which may be a consequence of the synaptic distribution variations of across the dendrites with fewer synapses located proximal than distal segments (Megías et al., 2001; Bloss et al., 2016). In agreement with this, the methodology implemented here provides the overall E/I ratios from whole synapse populations based on the use of several protein markers simultaneously.

Beyond E/I ratios, simultaneous imaging of 11 synaptic components enabled the identification and labeling of synapses based on synaptic content, and characterization of unique changes within these synapse subpopulations in response to chemical perturbation. Considering only those synapses that met the classification criteria for excitatory and inhibitory described here, we were able to identify five types of excitatory synapses and one type of inhibitory synapse based on the differential expression patterns of the targets specifically selected for this study. These synaptic targets, which are all major components of synapses, have been extensively studied for their role in synapse structure and function (Sheng and Hoogenraad, 2007; Sheng and Kim, 2011). Although the significance of these clusters may require additional studies because of the disproportionate complexity of the synaptic proteome (Husi et al., 2000; Peng et al., 2004; Collins et al., 2005, 2006; Emes and Grant, 2011; Distler et al., 2014). Notwithstanding, our analysis offers a framework for deciphering orchestrated compensatory changes that occur during synaptic scaling.

An additional layer of complexity that requires consideration, when classifying synapses using protein levels and correlation analysis, is the highly dynamic and plastic nature of synapses (Nimchinsky et al., 2002). Given this framework, the clusters we identified may represent synapses captured in various stages of remodeling, which may explain the diversity observed, especially regarding excitatory synapse subtypes. Another factor to consider is the influence of developmental factors on the morphologic, structural, and proteomic characteristics of synapses. Thus, an alternative interpretation of these clusters is that we captured synapses from various developmental stages.

We must also acknowledge the possibility that, because of the variation of antibody epitope specificity among different host species and purification methods, some of the identified clusters may indicate contamination from staining artifacts. While visual inspection of these synapses does not present any evidence to suggest this is the case, assessing detection of false positives could serve as a useful tool for improving accuracy.

Another interesting observation from our results is that excitatory synapses seem to be more heterogeneous than inhibitory synapses. Excitatory synapses present more diverse morphologies, which could account for the increased variation. However, we only incorporated two inhibitory markers, vGAT and gephyrin, which would limit the number of inhibitory clusters that could be detected. While these interpretations represent exciting possibilities for examination of synapse biology, further experimentation to test these hypotheses are critical to address all these different aspects associated with synapse diversity classification.

Another unexpected advantage of the platform implemented here is that it also provides a tool for the simultaneous examination of the population of dually innervated synapses, originally described by electron microscopy (Kubota et al., 2016; Villa et al., 2016). This population, observed here as positive for both vGlut1 and vGAT (12%), will likely require detailed characterization using super-resolution microscopy to overcome limitation of the spinning-disk confocal microscope used in this study (lateral resolution = 420 nm). Thus, this platform could be used in tandem with DNA-PRISM, which also has super-resolution capabilities (Guo et al., 2019).

Confocal imaging for each neuronal imaging experiment was conducted using a 63× objective with 1.15 numerical aperture. At the wavelengths imaged, we were able to examine large fields of view while also resolving smaller, synaptic structures above the diffraction limit. At this magnification it is impossible to ensure that all detected synapses are isolated, independent synaptic units. Nevertheless, within the confines of this common imaging limitation that is intrinsic to confocal, diffraction-limited imaging, PRISM multiplexing was still able to provide a significant improvement on conventional imaging because of its ability to resolve excitatory and inhibitory synapses simultaneously, and exclude dual synapses containing both markers from our analysis. In addition, by sampling a large number of 22,000 synapses, the number of individually resolved synapses should offer robust classification of the synaptic subtypes identified using multiplexed protein expression profiles.

Our results revealed differences in the responses to activity blockade at excitatory and inhibitory synapses. At excitatory synapses, the synaptic levels of postsynaptic scaffolding proteins and presynaptic neurotransmitter transporter levels increased whereas there were no changes in bassoon levels or changes in the presynaptic vesicle clustering protein synapsin1. Our results suggest that, at excitatory synapses, neurons respond to activity blockade by increasing the amount of neurotransmitter present per vesicle and increasing the available postsynaptic AMPA receptor binding slots, but not by increasing the number of synaptic vesicles that are available for release. In contrast, inhibitory synapses showed both an increase in the neurotransmitters amount in the vesicle and an increase in the reserve pool of vesicles, indicated by synapsin1. Synapsin1 has a critical function associated with the reserve pool of synaptic vesicles (Hilfiker et al., 1998; Bloom et al., 2003; Akbergenova and Bykhovskaia, 2007; Gitler et al., 2008), regulating mobilization of vesicles into the recycling pool (Chi et al., 2003; Cousin et al., 2003; Menegon et al., 2006; Baldelli et al., 2007), and forming clusters of vesicles that are reluctant to release unless high frequency stimulation is applied (Rizzoli and Betz, 2005). Increased synapsin1 at inhibitory synapses could therefore indicate reduced baseline GABAergic signaling consistent with the conventional model of synaptic scaling.

Synaptic diversity across various brain regions has been described previously using two postsynaptic markers PSD-95 and SAP102 (Zhu et al., 2018); however, a detailed characterization of synaptic diversity in cultured neurons and their response to changes in network activity with high-content imaging has not been addressed. An important component of homeostatic plasticity is that the resultant changes in excitability maintain the relative differences in synaptic strength between the individual synapses. Although no differences in the synapse diversity were observed in the presence of TTX treatment compared with control conditions; TTX was able to modify the clusters producing fewer synapses defined by low protein levels, consistent with the model that synaptic scaling induces global changes to increase synaptic strength. While this was true for synapses defined by our synaptic targets, there may be additional variations that require different targets for detection. While *in vitro* neuronal cultures lack the hierarchical organization and experience-driven plasticity that is present *in vivo*, *in vitro* models are effective tools for evaluating synapse development and plasticity quantitatively, as well as serving as a viable approach to perform large-scale drug and genetic screens. In the future, application of multiplexed imaging *in vivo* should offer increased power to assess synaptic heterogeneity, as well as reveal more physiologically relevant insight into neuronal plasticity.

In conclusion, PRISM facilitated the exploration of complex synaptic architecture, with the imaging and analysis platform described here revealing numerous synaptic subtypes and their molecular rearrangements in response to homeostatic scaling. Exploration of synaptic diversity in neurons enables a new range of investigations with the potential to reveal new connections between synaptic architecture and neuronal function that can be especially useful for high-content and high-throughput screening using compounds or siRNA libraries of genes associated with disease.

## Synthesis

Reviewing Editor: Alfonso Araque, University of Minnesota Decisions are customarily a result of the Reviewing Editor and the peer reviewers coming together and discussing their recommendations until a consensus is reached. When revisions are invited, a fact-based synthesis statement explaining their decision and outlining what is needed to prepare a revision will be listed below. The following reviewer(s) agreed to reveal their identity: Seth Grant, Gina Turrigiano.

Both reviewers consider satisfactory the responses to their concerns on the previous version. They are both very positive about the study and the manuscript in its present form. I concur with them.

Yet, there is one minor remaining issue that would need clarification before the manuscript is accepted. Could you please clarify it?

I am looking forward to receiving a final revised version of the manuscript including the clarification requested.

Sincerely,

Alfonso Araque

Reviewing Editor

eNeuro

Specific comments of the reviewers:

Reviewer 1.

This a very nice paper and advances our understanding of synapse molecular composition and neuronal activity. The methodological contributions are impressive and should stimulate considerable interest.

There is one minor remaining issue: The authors mentioned the effective pixel size is 187 nm. If the diffraction limit is also ∼187nm in xy, the imaged puncta would be quite small in the digital images (size ranging from 1x1 to 3x3 I guess) and therefore the puncta shape can hardly be measured. Would there be any typos in these values?

Reviewer 2.

The authors have addressed the concerns of the reviewers and the paper is now appropriate for publication

## Author Response

Responses to Reviewer 1:

Reviewer 1.

This a very nice paper and advances our understanding of synapse molecular composition and neuronal activity. The methodological contributions are impressive and should stimulate considerable interest.

There is one minor remaining issue: The authors mentioned the effective pixel size is 187 nm. If the diffraction limit is also ∼187nm in xy, the imaged puncta would be quite small in the digital images (size ranging from 1x1 to 3x3 I guess) and therefore the puncta shape can hardly be measured. Would there be any typos in these values?

We thank the reviewer for their careful ongoing review of our work and we appreciate their positive feedback. The text was indeed incorrect; the numerical pixel size was mentioned instead of the microscope lateral resolution, which is in fact 420nm. The corresponding sentence has been corrected and the revised text now reads, “...will likely require detailed characterization using super-resolution microscopy to overcome limitation of the spinning-disk confocal microscope used in this study (lateral resolution=420nm)"

Additionally, a size restriction step was part of the object detection portion of the analysis pipeline. We have, accordingly, modified the methods section to empathize this point and now reads, “Only puncta with equivalent diameters between 3-15 pixels were included in the analysis, where the equivalent diameter was defined by a circle of the same area as the segmented object”.

Reviewer 2.

The authors have addressed the concerns of the reviewers and the paper is now appropriate for publication
