# Supplementary figures and images for: Molecular Diversity of Glutamatergic and GABAergic Synapses from Multiplexed Fluorescence Imaging
Source: eNeuro. 2021 Jan 15;8(1):ENEURO.0286-20.2020. doi: 10.1523/ENEURO.0286-20.2020 (PMC7877457; doi:10.1523/ENEURO.0286-20.2020)

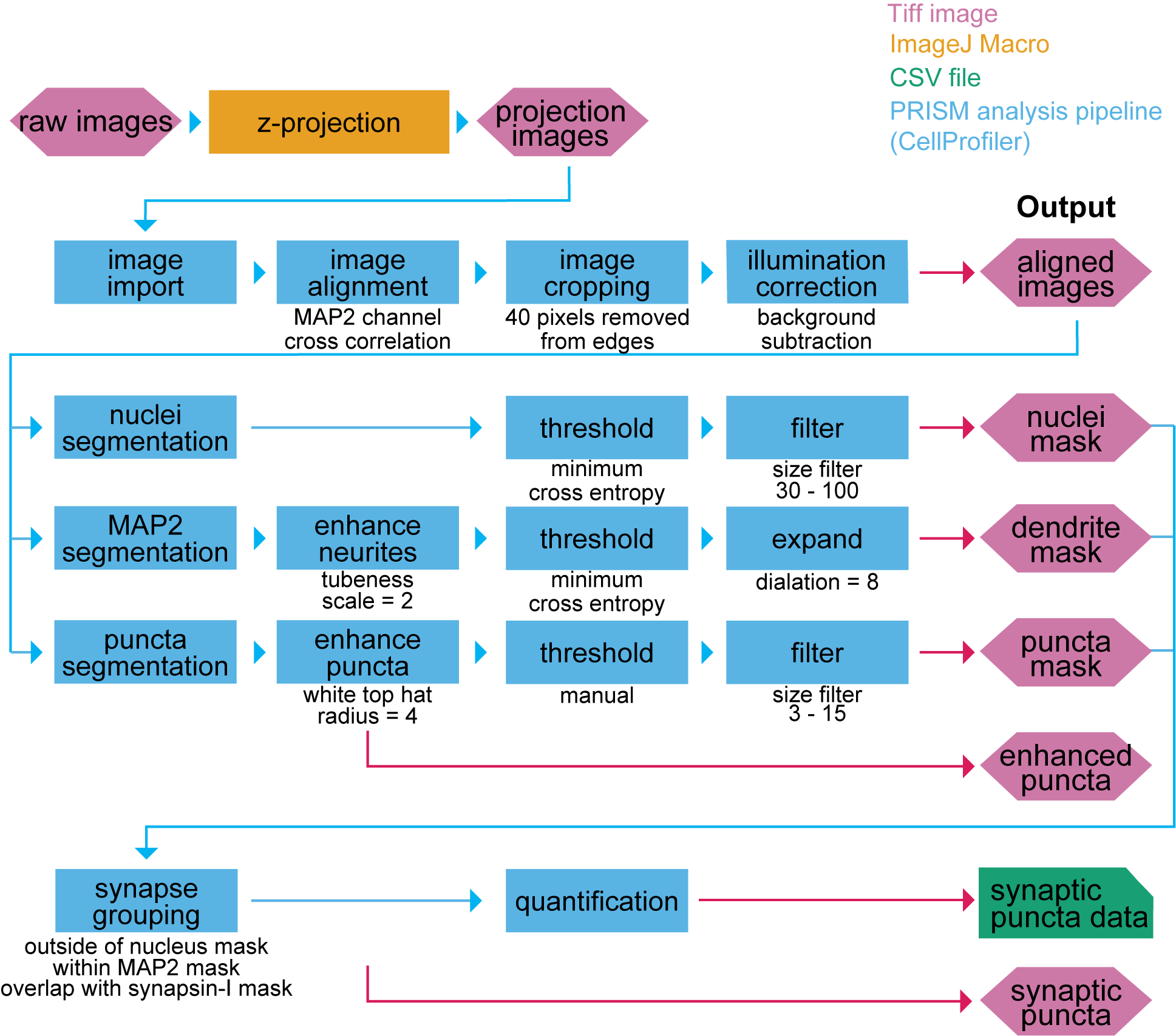

Supplement: Extended Data Figure 1-1 — Overview of CellProfiler workflow. The CellProfiler pipeline takes maximum projection images as input then aligns the images and performs illumination correction by subtracting background signal. The pipeline next defines nuclei objects using DAPI, dendrite objects, using MAP2 and puncta objects using the various PRISM and IF staining. In the last stage of the pipeline, the puncta objects are organized into synapses. Puncta objects are considered synaptic if the objects are outside of the nuclei, are within eight pixels of the dendritic mask and are overlapping with synapsin1 objects. Data are exported to a csv file (green) and multiple images (purple) are created during each stage for quality control. Download Figure 1-1, TIF file. [file enu-eN-MNT-0286-20-s03.tif]

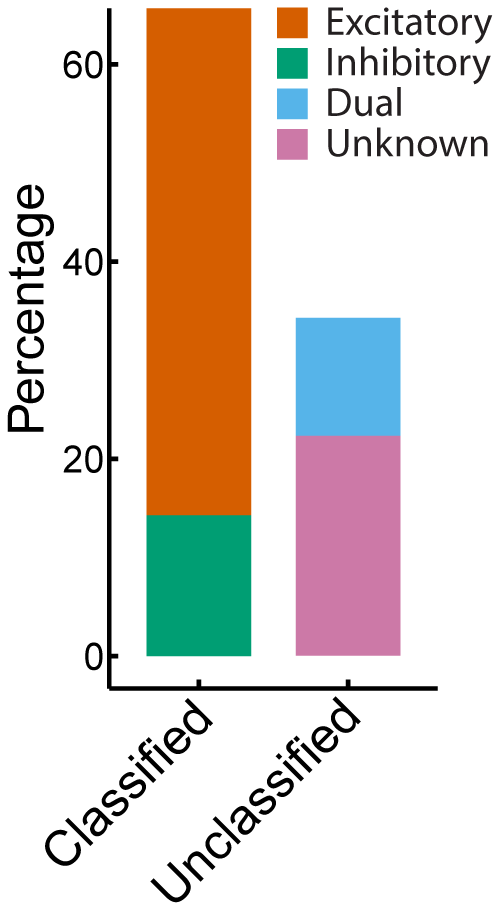

Supplement: Extended Data Figure 1-3 — Classification of synapses using CellProfiler. Synapses are classified based on the presence of synapsin1, vGlut1, and vGAT. Synapses with synapsin1 and only vGlut1 are classified as excitatory. Synapses with synapsin1 and only vGAT are classified as inhibitory. The remaining synapses are unclassified and contain either synapsin1 alone, or synapsin1 and both vGlut1 and vGAT. Download Figure 1-3, TIF file. [file enu-eN-MNT-0286-20-s04.tif]

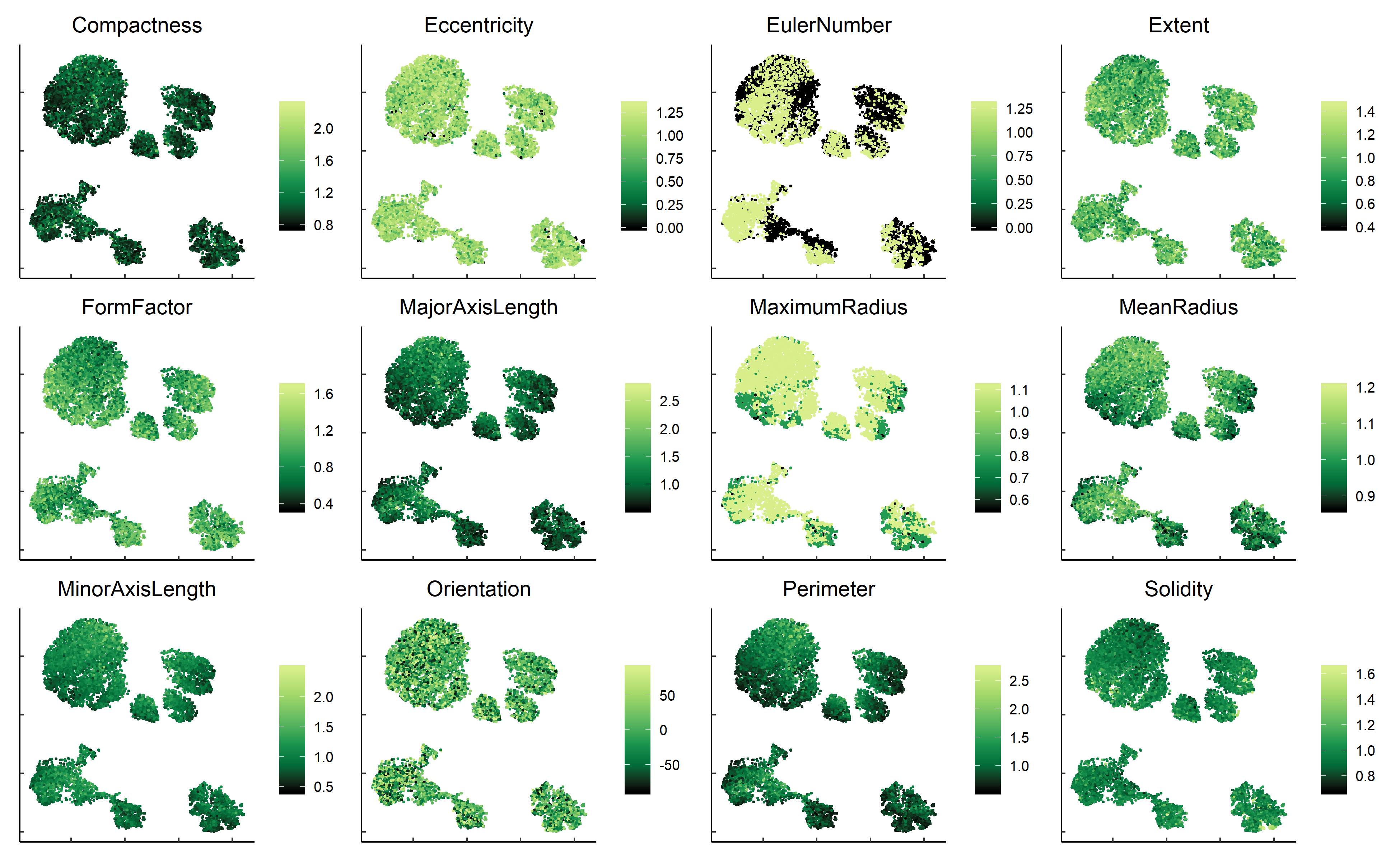

Supplement: Extended Data Figure 2-1 — UMAP analysis of synapsin1 shape measurements. UMAP analysis of synapses colored for indicated synapsin1 shape metrics. Download Figure 2-1, TIF file. [file enu-eN-MNT-0286-20-s12.tif]

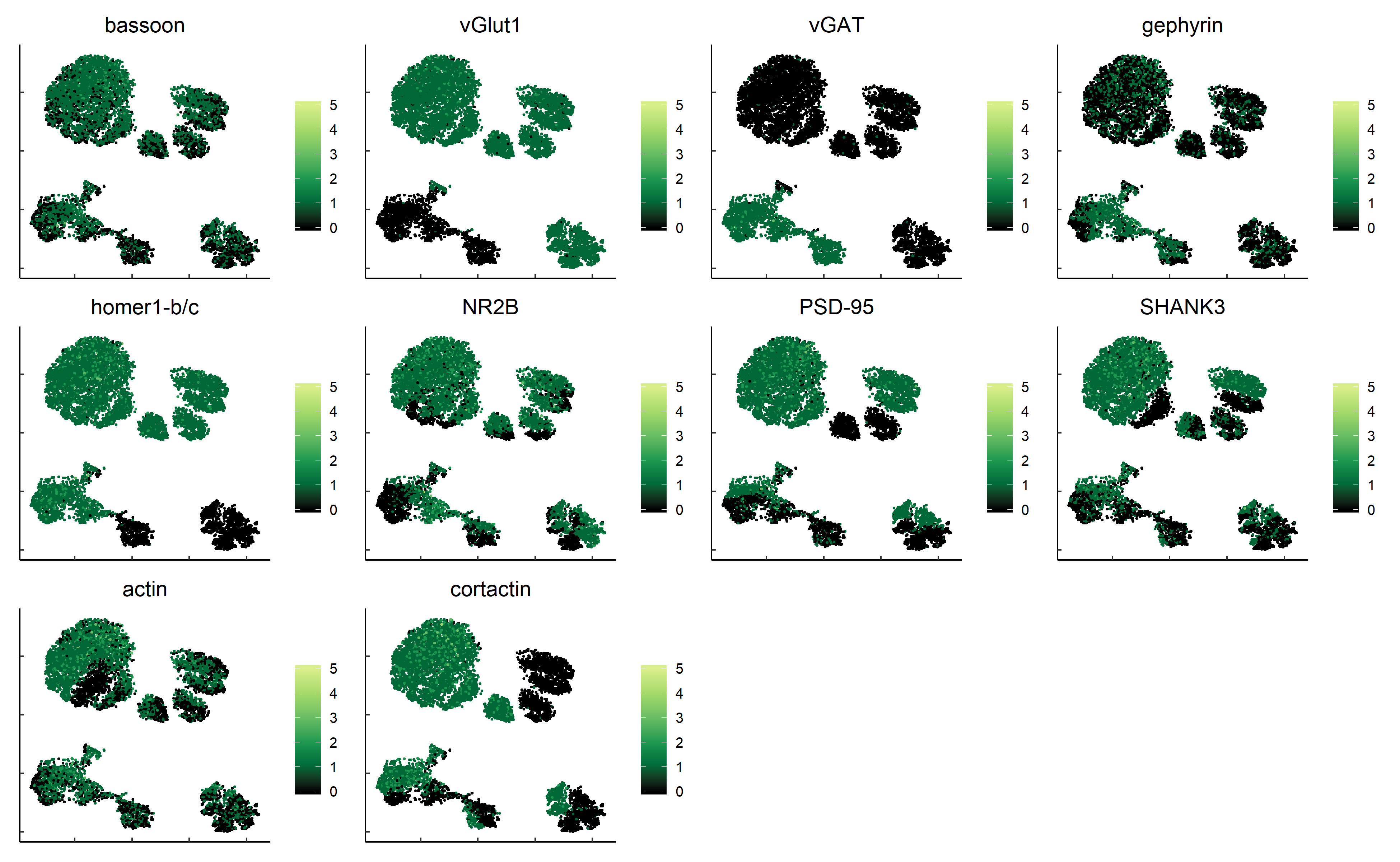

Supplement: Extended Data Figure 2-2 — UMAP analysis of puncta number per synapse. UMAP analysis shows synapses colored for the number of puncta per synapse for each synaptic target. Download Figure 2-2, TIF file. [file enu-eN-MNT-0286-20-s05.tif]

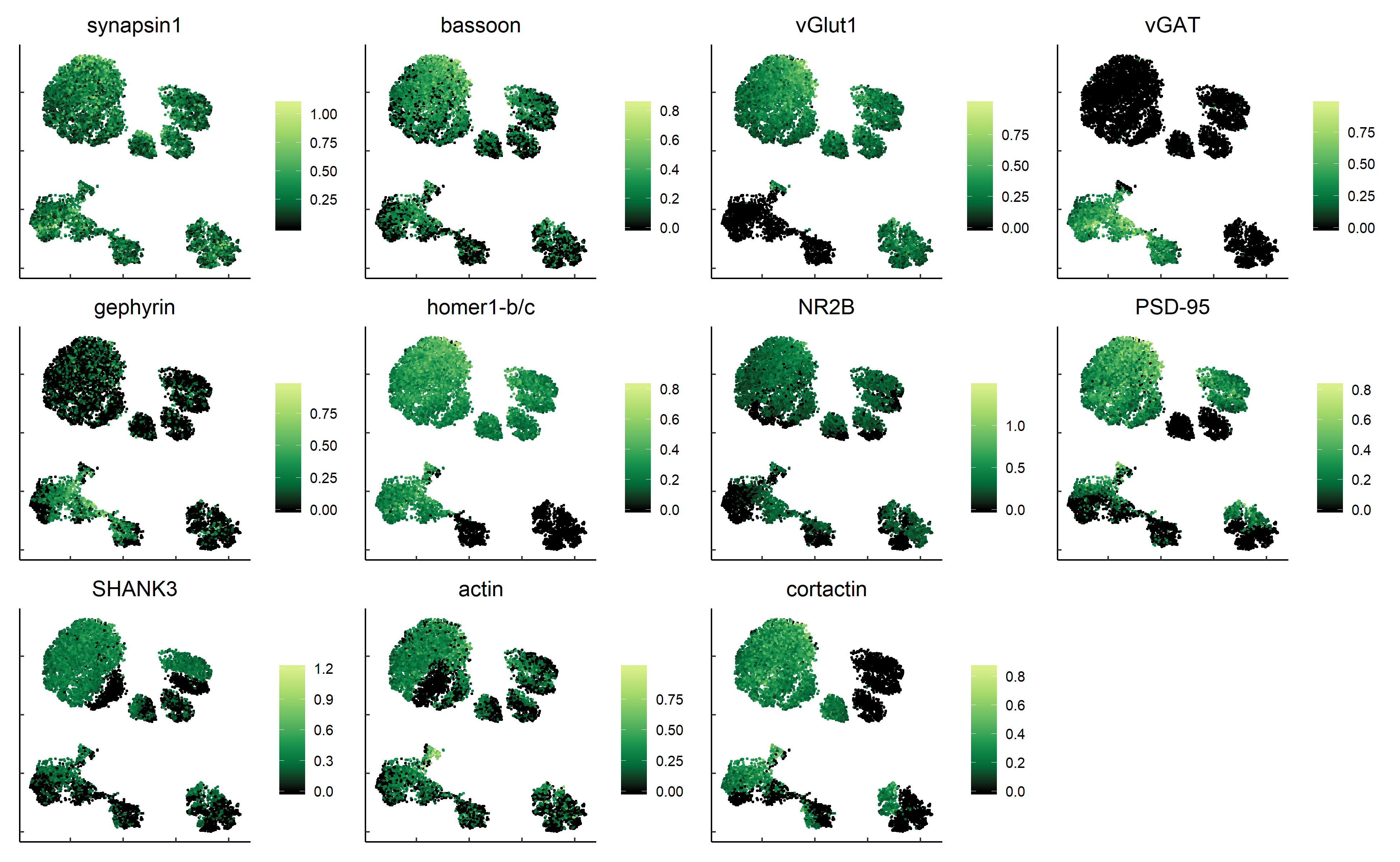

Supplement: Extended Data Figure 2-3 — UMAP analysis of relative integrated intensity. UMAP analysis of synapses colored for relative integrated intensity of each synaptic target. Scale bar shows log values. Download Figure 2-3, TIF file. [file enu-eN-MNT-0286-20-s06.tif]

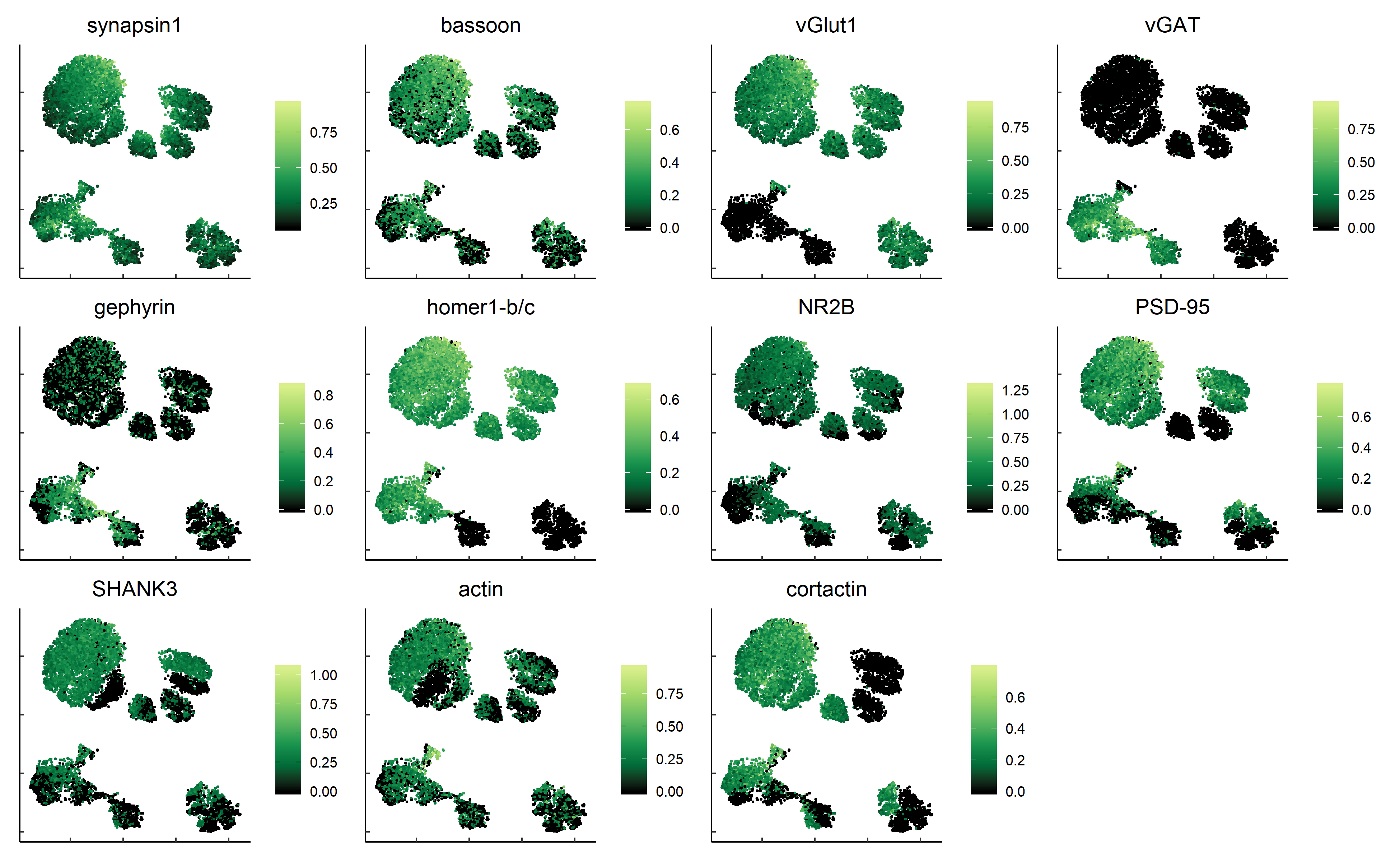

Supplement: Extended Data Figure 2-4 — UMAP analysis of relative integrated intensity of puncta edge. UMAP analysis of synapses colored for relative integrated intensity of the edge of each punctum for each synaptic target. Scale bar show log values. Download Figure 2-4, TIF file. [file enu-eN-MNT-0286-20-s07.tif]

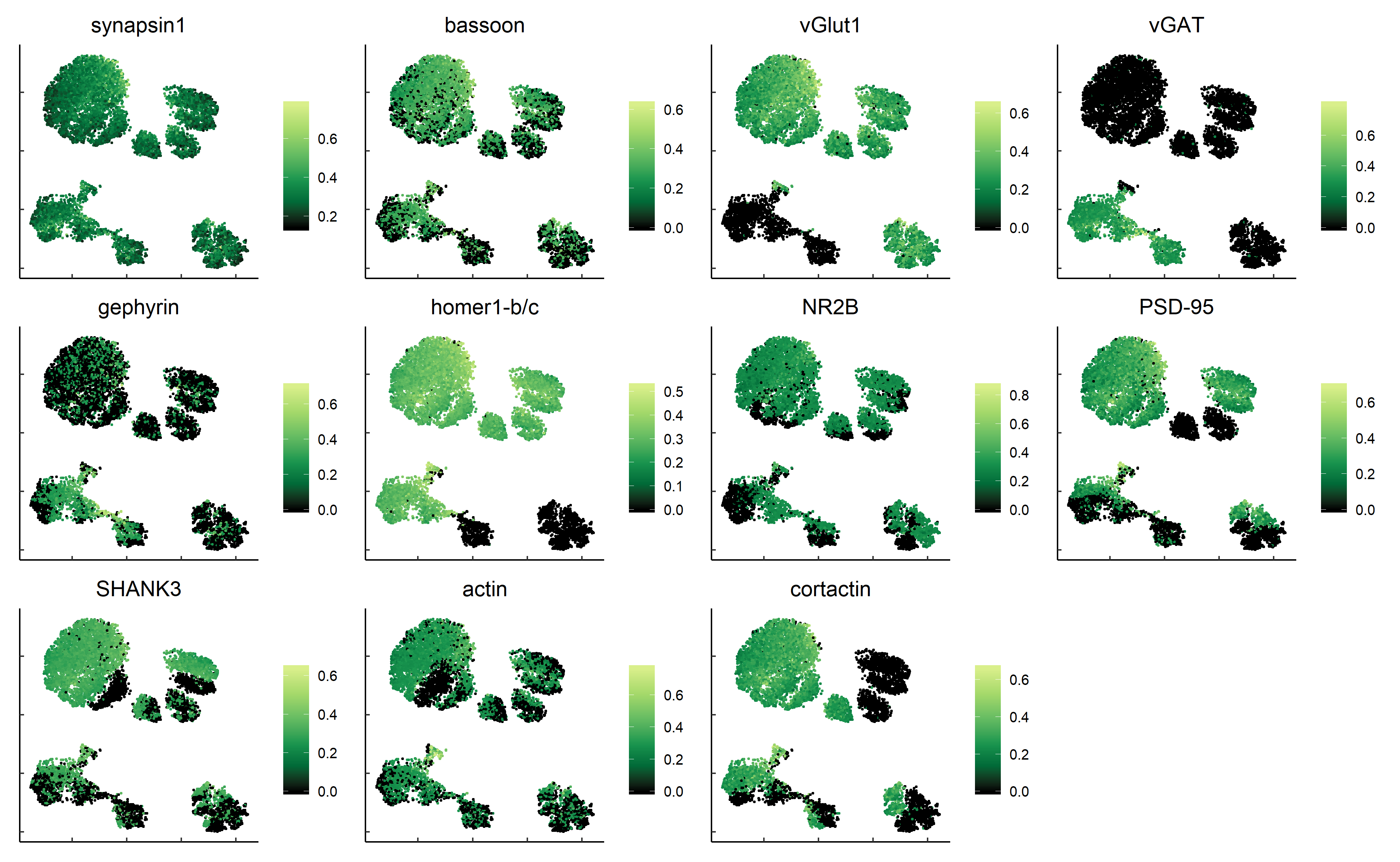

Supplement: Extended Data Figure 2-5 — UMAP analysis of lower quartile intensity. UMAP analysis of synapses colored for the lower quartile intensity (log values) of each punctum for each synaptic target. Download Figure 2-5, TIF file. [file enu-eN-MNT-0286-20-s08.tif]

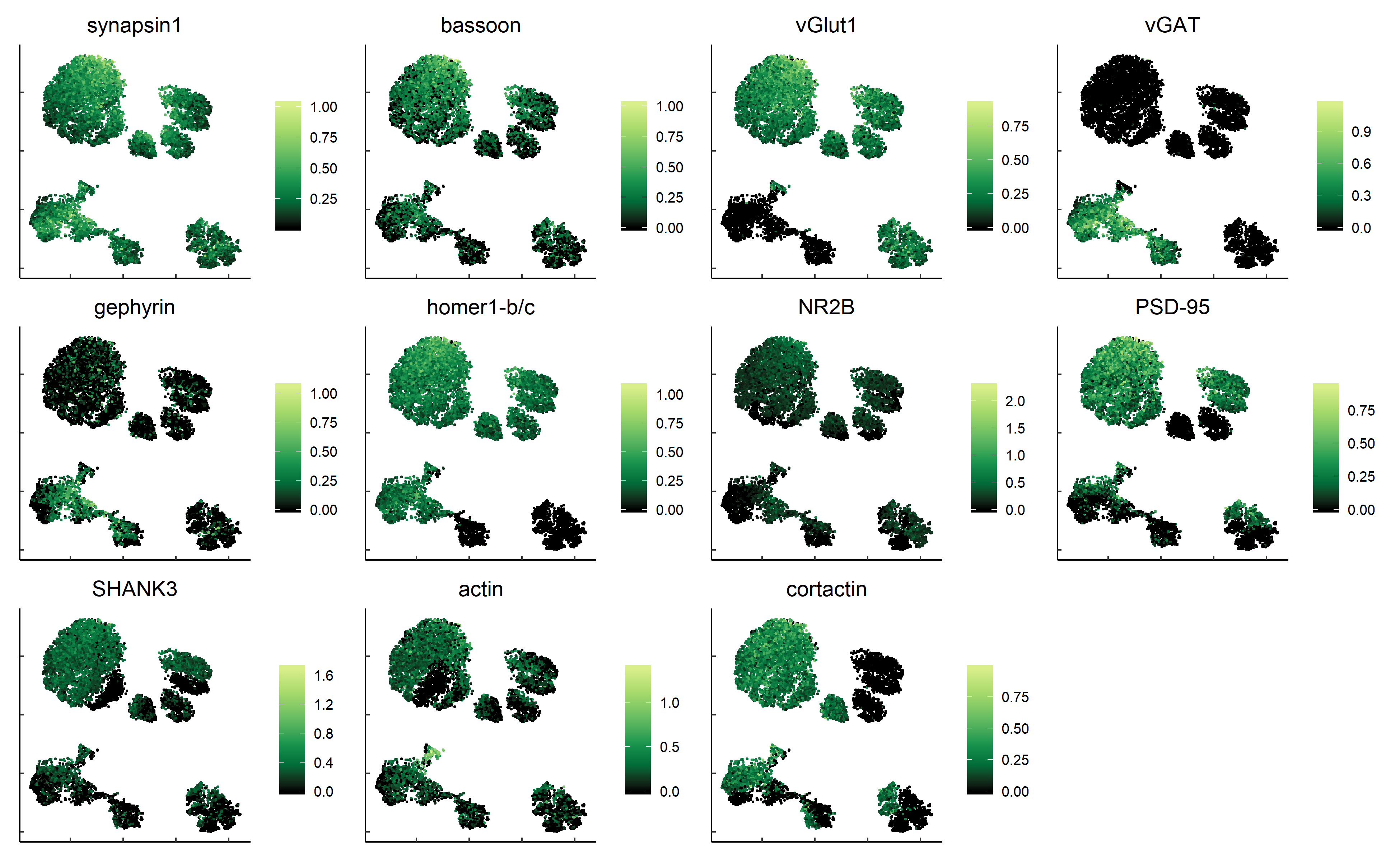

Supplement: Extended Data Figure 2-6 — UMAP analysis of median absolute deviation. UMAP analysis of synapses colored for median absolute deviation intensity (log values) of each punctum for each synaptic target. Download Figure 2-6, TIF file. [file enu-eN-MNT-0286-20-s09.tif]

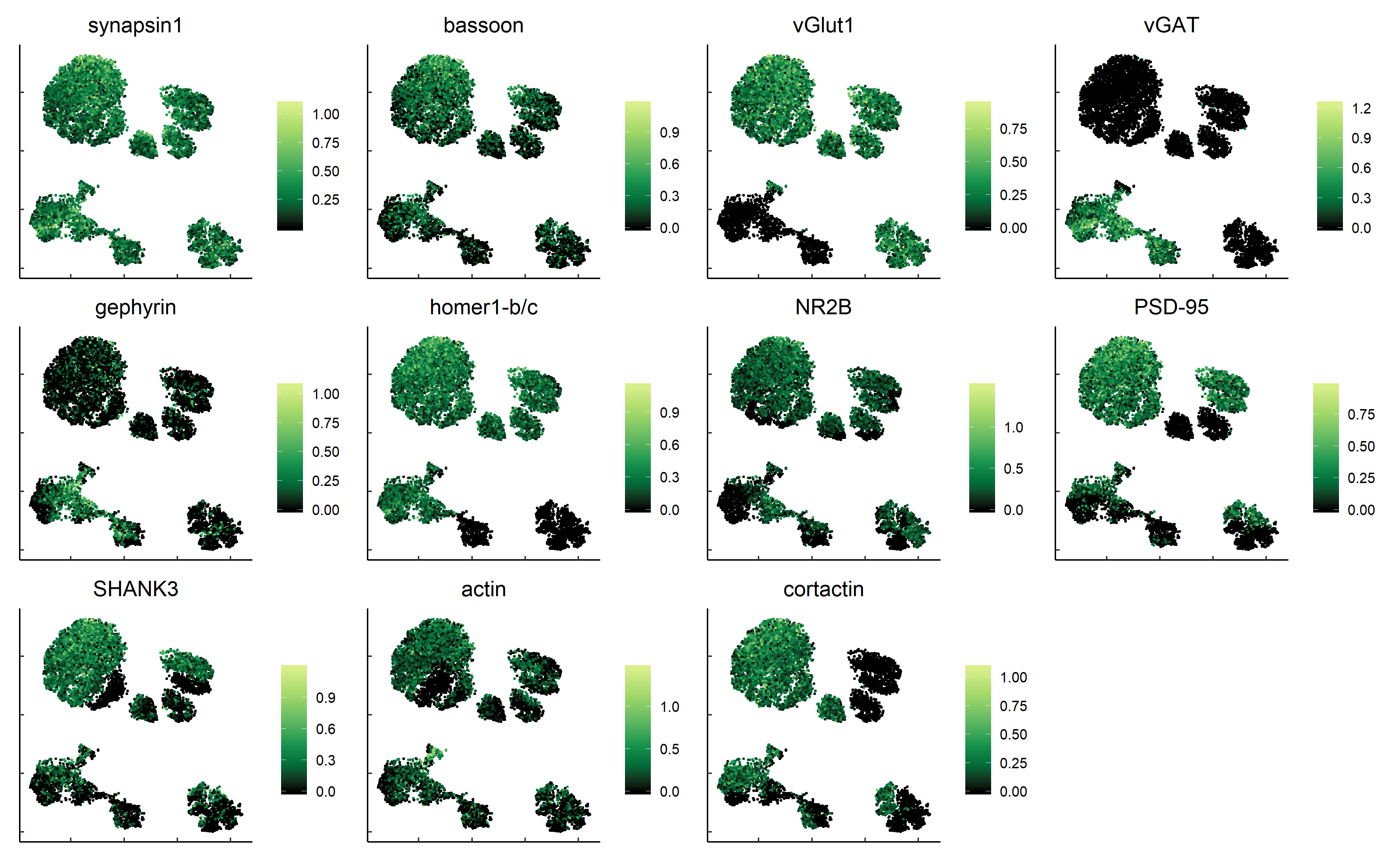

Supplement: Extended Data Figure 2-7 — UMAP analysis of puncta mass displacement. UMAP analysis of synapses colored for the mass displacement (log values) of each punctum for each synaptic target. Download Figure 2-7, TIF file. [file enu-eN-MNT-0286-20-s10.tif]

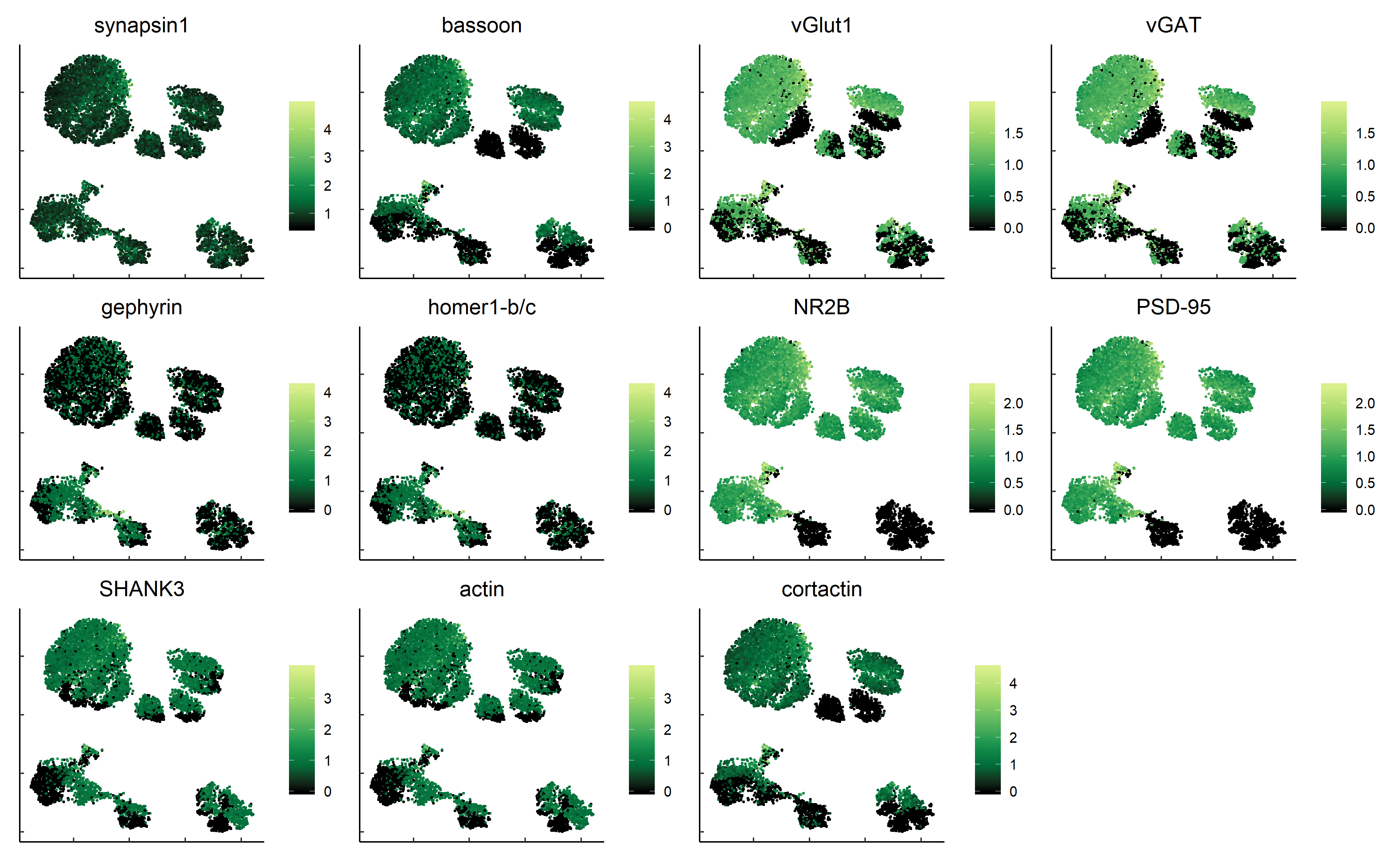

Supplement: Extended Data Figure 2-8 — UMAP analysis of minimum pixel intensity of puncta edge. UMAP analysis of synapses colored for minimum pixel intensity on the edge of each punctum for each synaptic target. Download Figure 2-8, TIF file. [file enu-eN-MNT-0286-20-s11.tif]

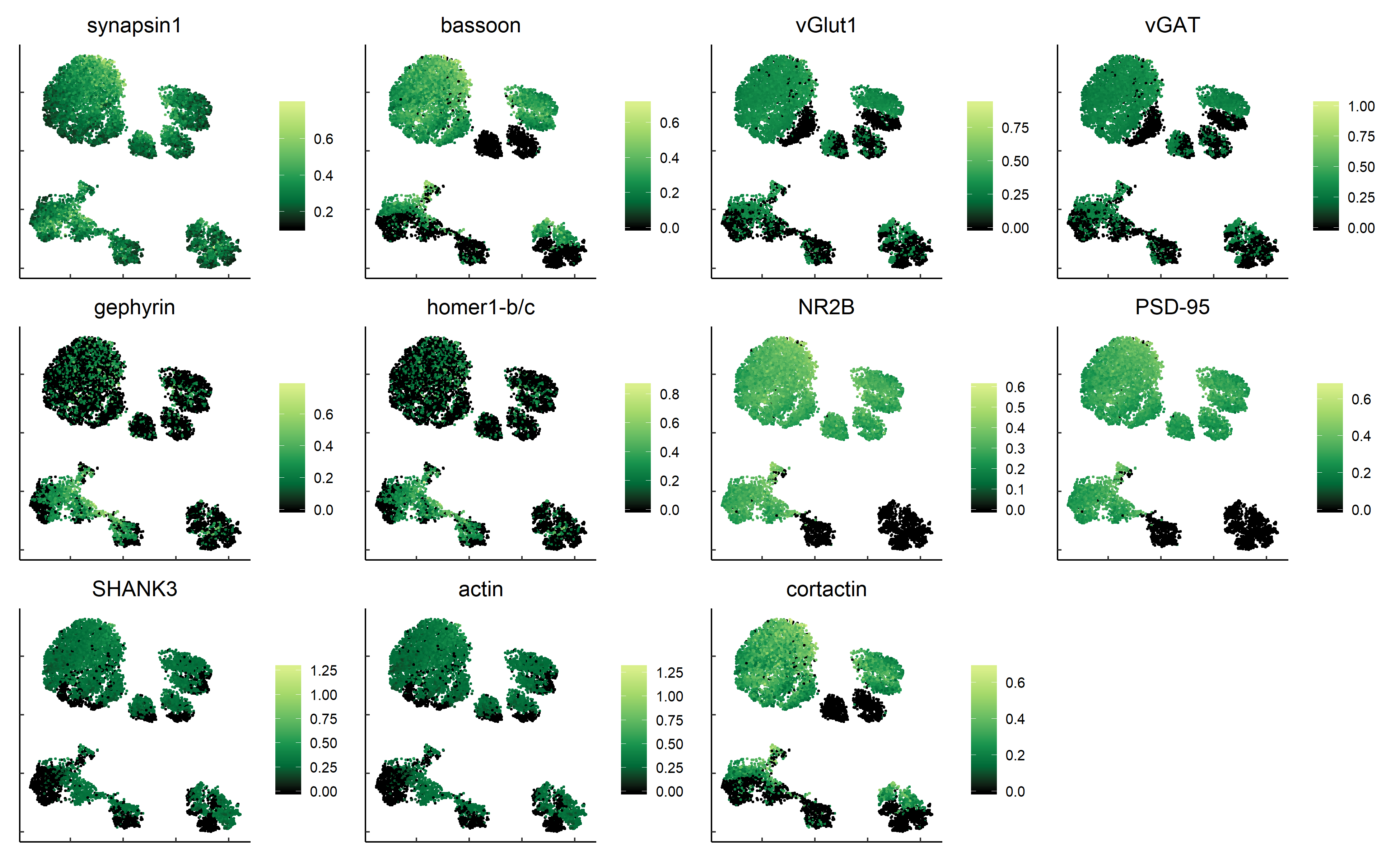

Supplement: Extended Data Figure 2-9 — UMAP analysis of puncta maximum intensity. UMAP analysis of synapses colored for maximum intensity (log values) of each punctum for each synaptic target. Download Figure 2-9, TIF file. [file enu-eN-MNT-0286-20-s13.tif]

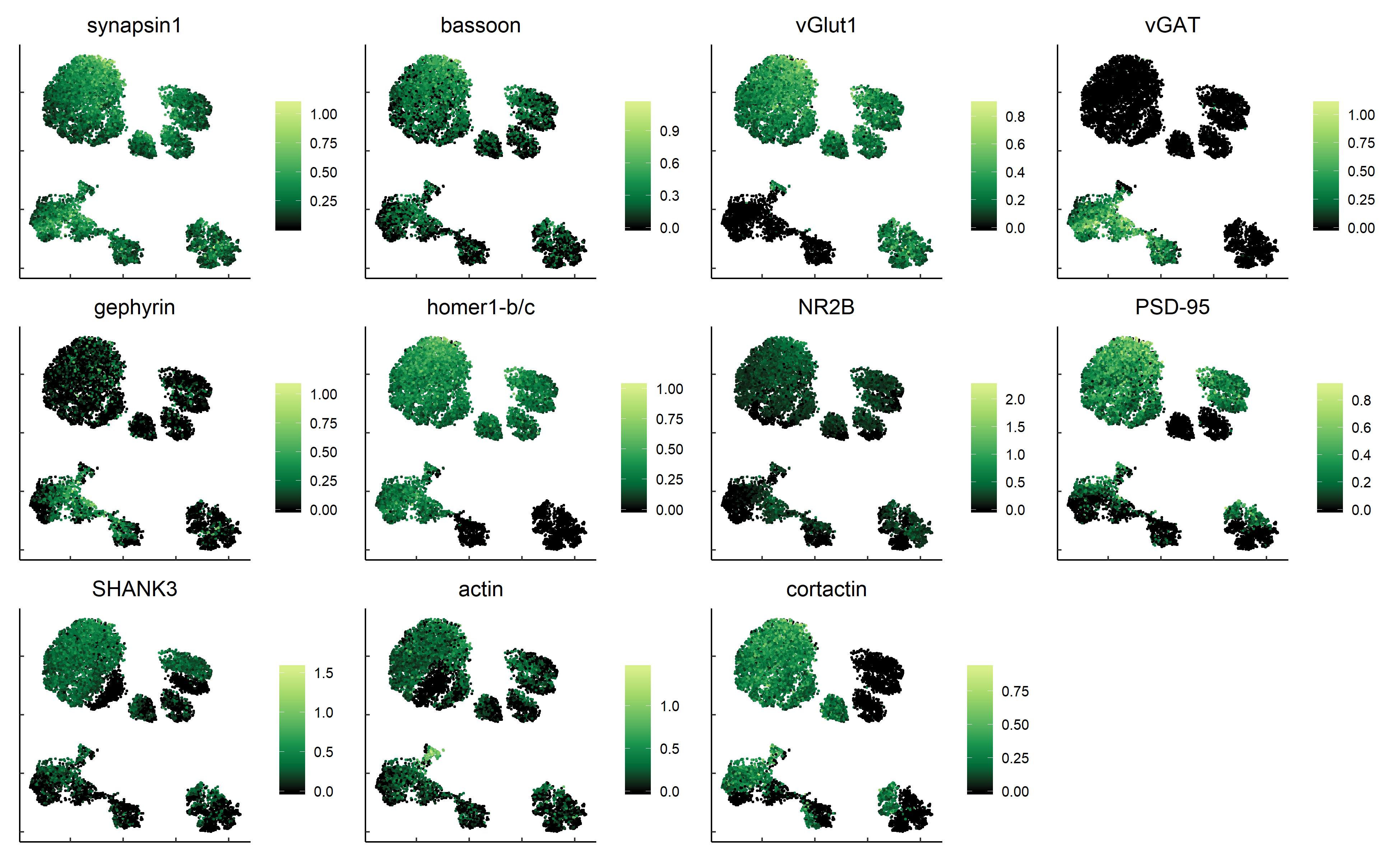

Supplement: Extended Data Figure 2-10 — UMAP analysis of puncta edge standard deviation. UMAP analysis of synapses colored for standard deviation of the edge (log values) of each punctum for each synaptic target. Download Figure 2-10, TIF file. [file enu-eN-MNT-0286-20-s14.tif]

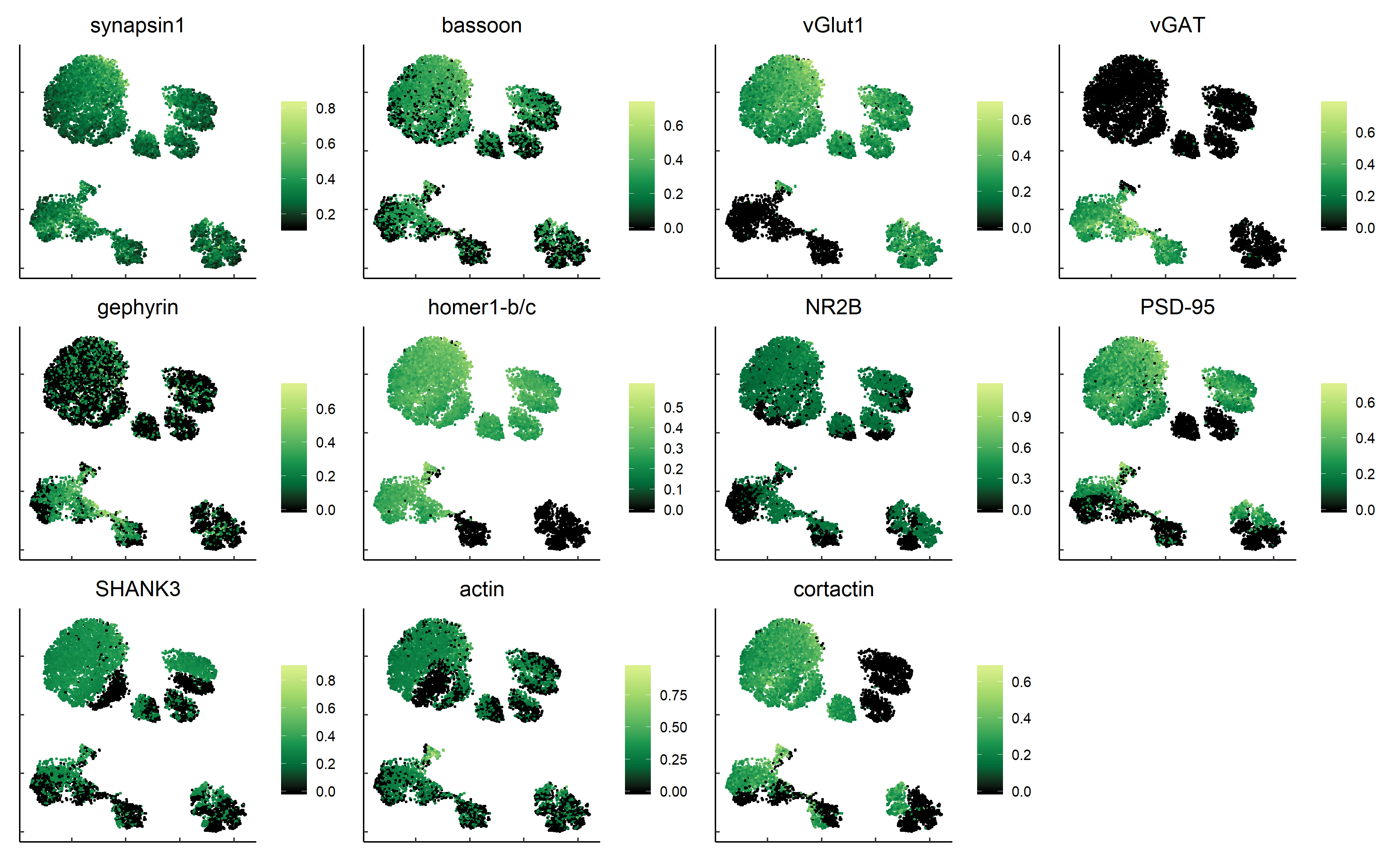

Supplement: Extended Data Figure 2-11 — UMAP analysis of puncta upper quartile intensity. UMAP analysis of synapses colored for the upper quartile intensity of each punctum for each synaptic target. Download Figure 2-11, TIF file. [file enu-eN-MNT-0286-20-s15.tif]

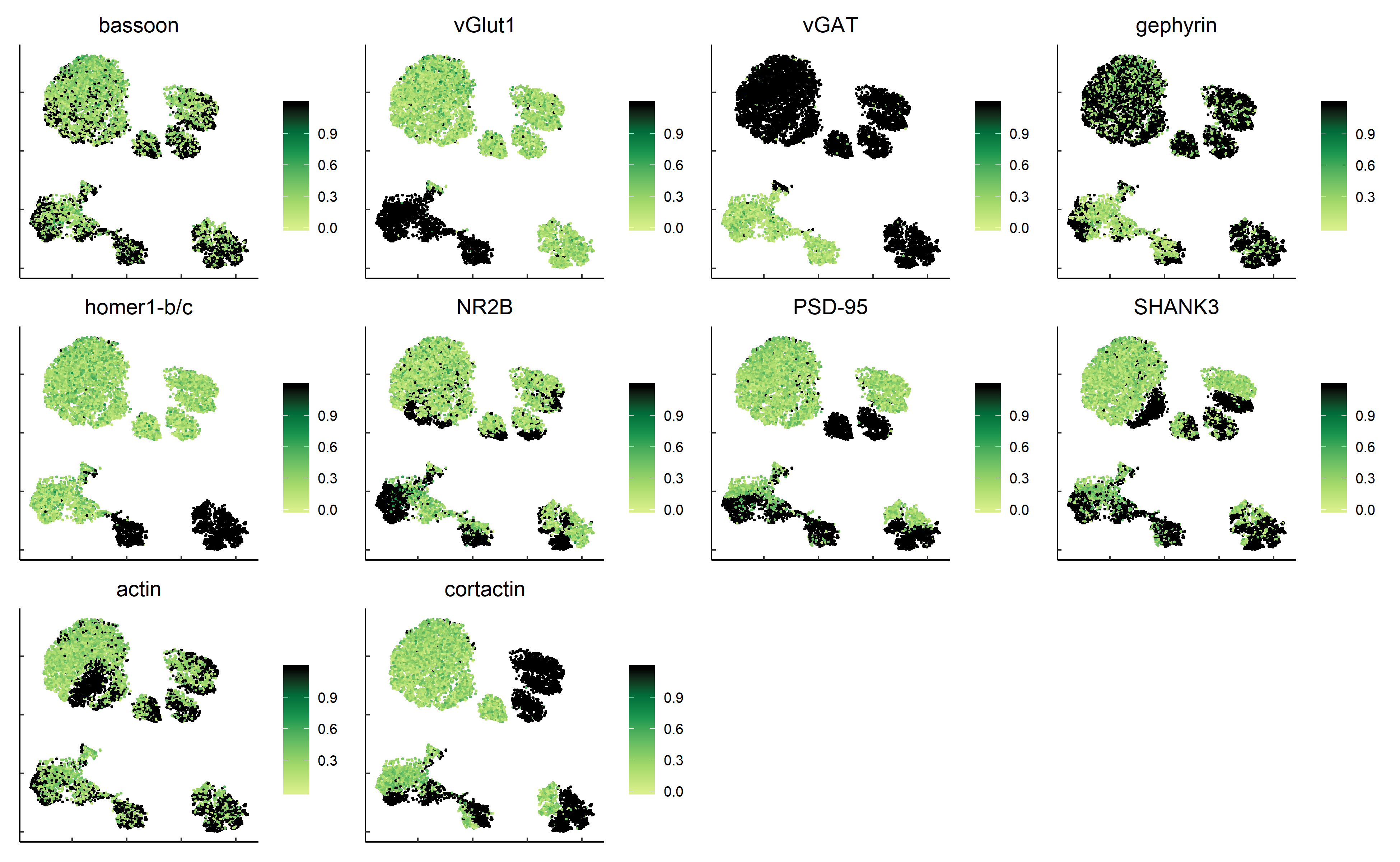

Supplement: Extended Data Figure 2-12 — UMAP analysis of distance to synapsin1 puncta centroid. UMAP analysis of synapses colored for distance in pixels (log value) between puncta centroids (synapsin1 and each indicated target). Download Figure 2-12, TIF file. [file enu-eN-MNT-0286-20-s16.tif]

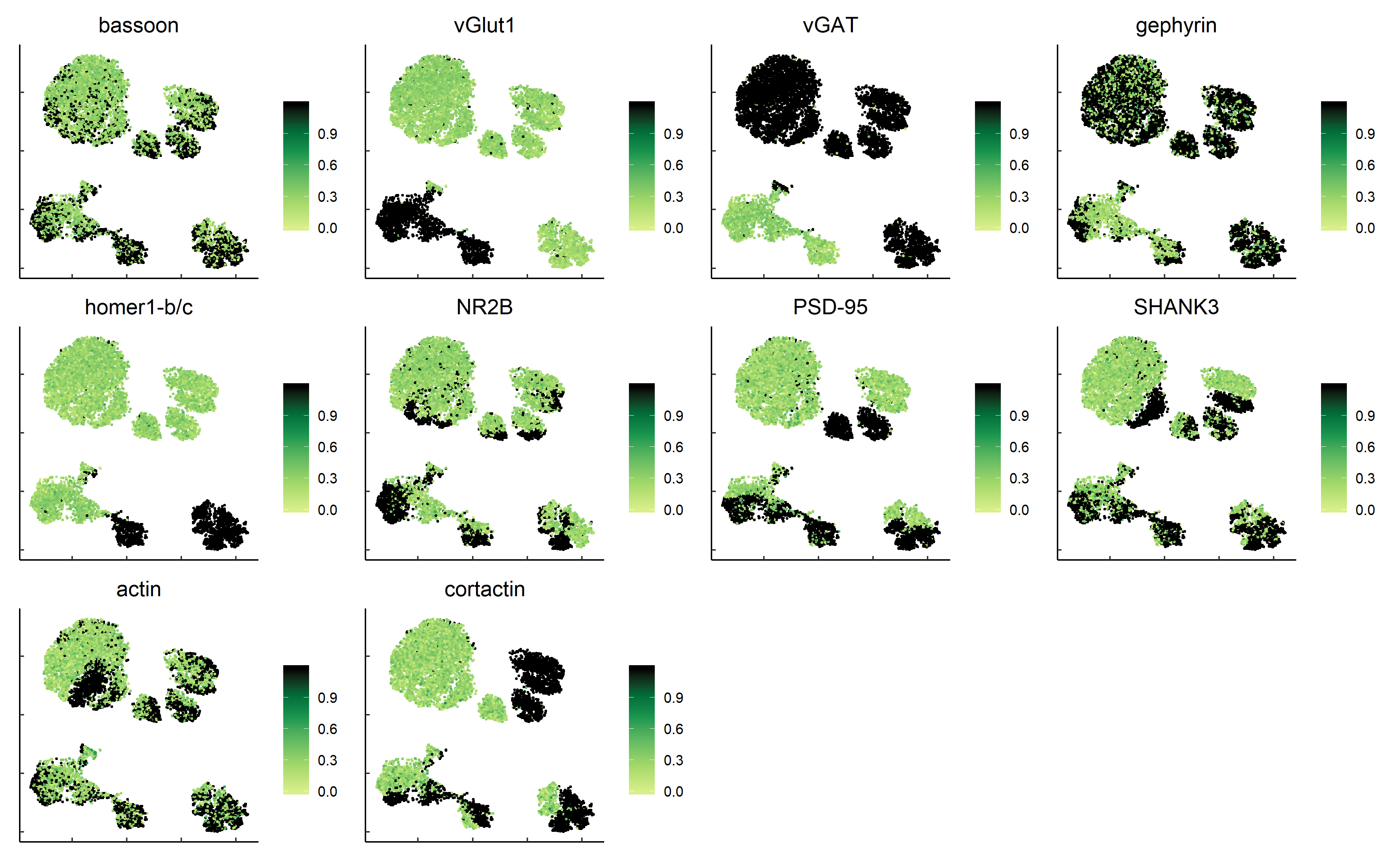

Supplement: Extended Data Figure 2-13 — UMAP analysis of minimum distance to synapsin1 puncta. UMAP analysis of synapses colored for minimum distance in pixels (log values) between the indicated target and synapsin1. Download Figure 2-13, TIF file. [file enu-eN-MNT-0286-20-s17.tif]

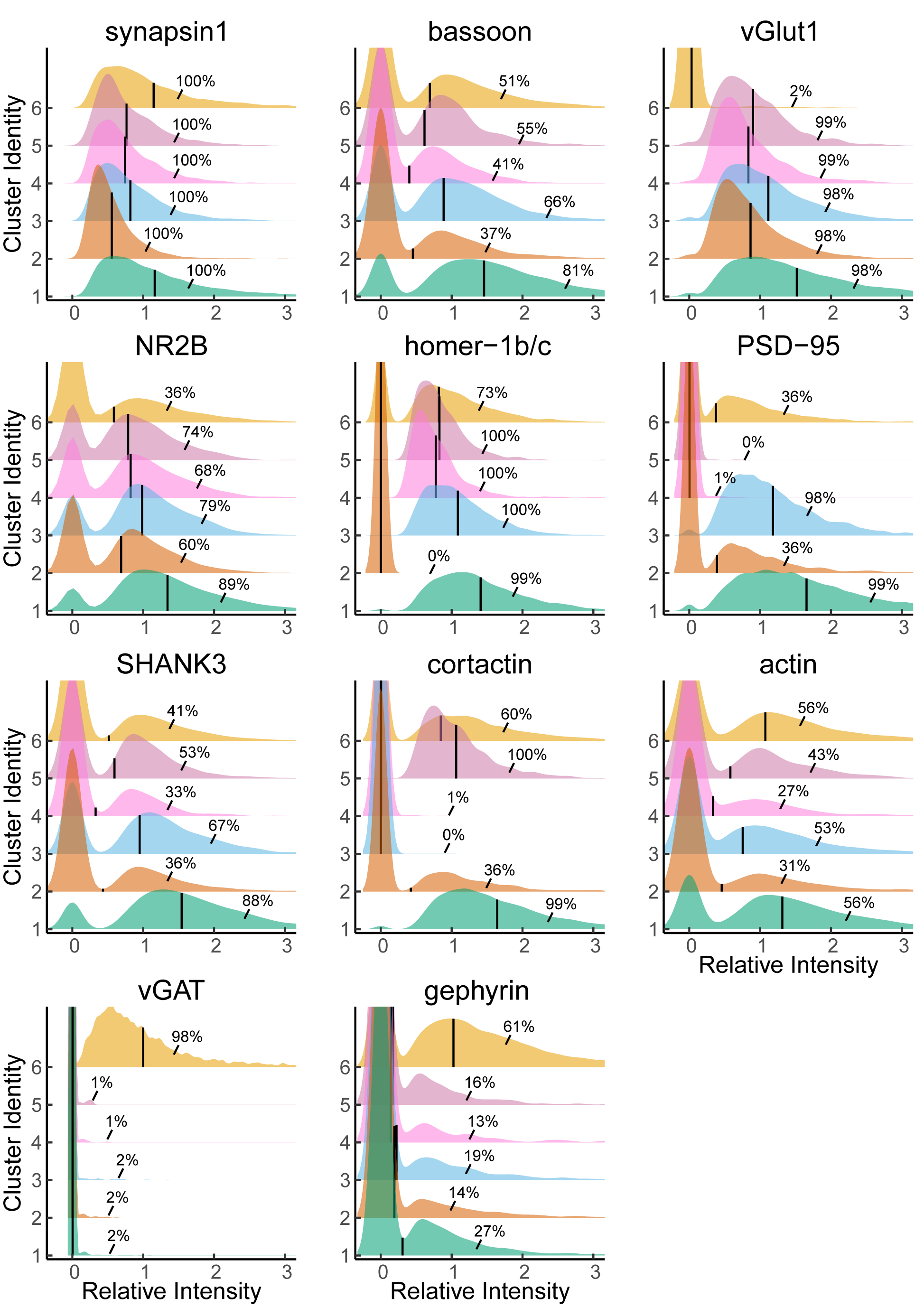

Supplement: Extended Data Figure 2-14 — Relative synaptic intensities of all targets for each synaptic subtype. Ridgeline plots of relative synaptic intensities for synaptic targets within cluster groups identified using HDBScan. Horizontal black line represents cluster mean intensity. All values are normalized to untreated mean integrated intensity. Percent of synapses with integrated intensity >0 for each cluster. Download Figure 2-14, TIF file. [file enu-eN-MNT-0286-20-s18.tif]

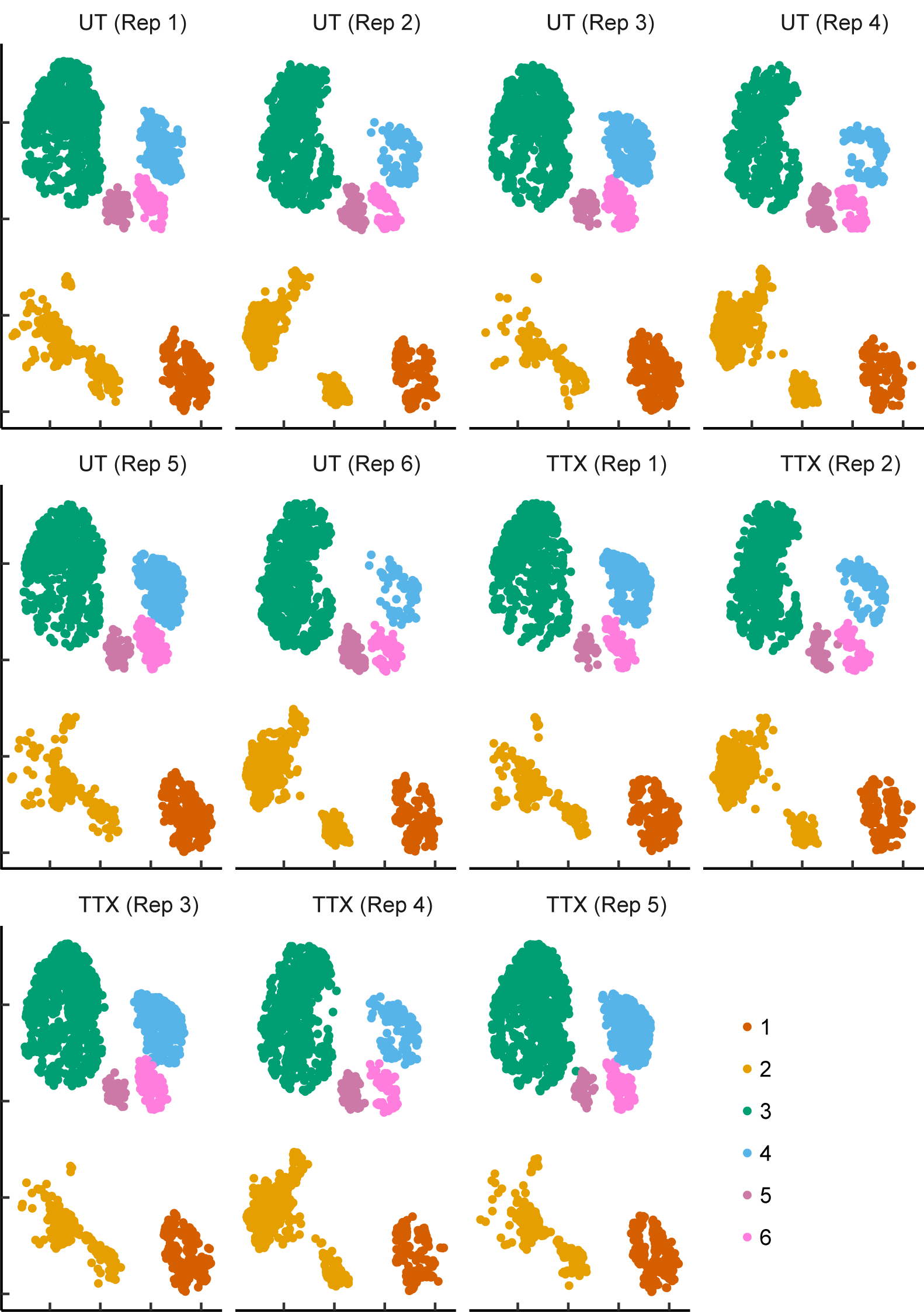

Supplement: Extended Data Figure 5-1 — Synaptic cluster identification for individual replicates. UMAP plots of individual synapses using CellProfiler output separates excitatory and inhibitory synapses into approximately six unique clusters identified by HDBSCAN. Each cluster is present in each sample indicating unique cluster formation is not an artifact of individual differences in culture conditions across different samples. Download Figure 5-1, TIF file. [file enu-eN-MNT-0286-20-s19.tif]
