# Supplementary material for: Molecular Diversity of Glutamatergic and GABAergic Synapses from Multiplexed Fluorescence Imaging
Source: eNeuro. 2021 Jan 15;8(1):ENEURO.0286-20.2020. doi: 10.1523/ENEURO.0286-20.2020 (PMC7877457; doi:10.1523/ENEURO.0286-20.2020)
Supplement: Extended Data Figure 1-2 — Synaptic properties measured by CellProfiler. The various intensity, shape, distance and object number measurements performed by CellProfiler. Each measurement is performed on each target from PRISM staining. Download Figure 1-2, PDF file. [file enu-eN-MNT-0286-20-s02.pdf]

Figure 1-2 Synaptic properties measured by CellProfiler.

**Type**	**Name**	**Description**
Intensity	Integrated intensity	The sum of the pixel intensities of an object
	Mean intensity	The average pixel intensity of an object
	Std intensity	The standard deviation of the pixel values of an object
	Max intensity	The maximal pixel intensity of an object
	Min intensity	The minimal pixel intensity of an object.
	Integrated intensity edge	The sum of the edge pixel intensities of an object.
	Mean intensity edge	The average edge pixel intensity of an object
	Std intensity edge	The standard deviation of the edge pixel values of an object
	Max intensity edge	The maximal edge pixel intensity of an object
	Min intensity edge	The minimal edge pixel intensity of an object
	Mass displacement	The distance between the centers of gravity in the gray-level and the binary representations of the object

	Lower quartile intensity	The intensity value of the pixel for which 25% of the pixels in the object have lower values
	Median intensity	The median intensity value within the object
	MAD intensity	The median absolute deviation (MAD), defined as the median ($\|x_i - \text{median}(x)\|$), of the intensities in the object
	Upper quartile intensity	The intensity value of the pixel for which 75% of the pixels in the object have lower values
Shape	Area	The number of pixels in the object
	Perimeter	The number of pixels on the boundary of each object
	Form factor	4π X Area / Perimeter^2
	Solidity	The proportion of pixels in both the convex hull and object
	Extent	The proportion of pixels in the bounding box and the object
	Euler number	The contained object number minus the number of holes
	Eccentricity	The ellipse eccentricity with the same second-moment

	Axis length (major, minor)	The length of the major/minor axis of the ellipse with the same normalized second central moments as the object
	Orientation	The angle between the x-axis and the major axis of the ellipse with the same second-moments as the object
	Compactness	The mean squared distance of the object's pixels from the centroid divided by the area.
	Maximum radius	The maximum distance of any pixel in the object to the closest pixel outside of the object
	Median radius	The median distance of any pixel in the object to the closest pixel outside of the object
	Mean radius	The mean distance of any pixel in the object to the closest pixel outside of the object
	Feret diameter (min,max)	The distance between two parallel lines tangent on either side of the object
Distance	Centroid	The distance between the centroids of two objects
	Minimum	The minimum distance between two objects
Object number	Children	The number of objects of that overlap with another object
